# The Development of Highly Specific and Sensitive Primers for the Detection of Potentially Allergenic Soybean (*Glycine max*) Using Loop-Mediated Isothermal Amplification Combined with Lateral Flow Dipstick (LAMP-LFD)

**DOI:** 10.3390/foods9040423

**Published:** 2020-04-03

**Authors:** Stefanie M. Allgöwer, Chris A. Hartmann, Thomas Holzhauser

**Affiliations:** Division of Allergology, Paul-Ehrlich-Institut (PEI), D-63225 Langen, Germany; stefanie.allgoewer@arcor.de (S.M.A.); chris.a.hartmann@gmx.de (C.A.H.)

**Keywords:** multicopy gene, rapid test, loop-mediated isothermal amplification, LAMP, lateral flow dipstick, LFD, food allergy, allergen detection, soybean, *Glycine max*

## Abstract

The soybean (*Glycine max*) has been recognized as a frequent elicitor of food allergy worldwide. A lack of causative immunotherapy of soybean allergy makes soybean avoidance essential. Therefore, sensitive and specific methods for soybean detection are needed to allow for soybean verification in foods. Loop-mediated isothermal amplification (LAMP) represents a rapid and simple DNA-based detection method principally suitable for field-like applications or on-site analytical screening for allergens during the manufacturing of foods. This work describes the systematic development and selection of suitable LAMP primers based on soybean multicopy genes. The chemistry applied allows for a versatile detection of amplified DNA, using either gel electrophoresis, fluorescence recording, or a simple Lateral Flow Dipstick (LFD). LAMP based on the *ORF160b* gene was highly specific for the soybean and may allow for a detection level equivalent to approximately 10 mg soy per kg food. Various soybean cultivars were detectable at a comparable level of sensitivity. LAMP combined with LFD-like detection facilitates a simple, highly specific and sensitive detection of the soybean without the need for expensive analytical equipment. In contrast to the majority of antibody-based methods for soybean detection, all identified primer sequences and optimized protocols are disclosed and broadly available to the community.

## 1. Introduction

The soybean is a member of the *Fabaceae* botanical family. It is widely used in the food market, due to its nutritional value and functional properties in food products, as a texturizer, emulsifier, and protein filler, as well as for the production of meat analogues and milk substitutes [1,2]. However, soybeans are also known to cause food allergy, which is an abnormal immune response to food proteins [3]. Symptoms of soybean allergy may range from mild to severe systemic reactions including anaphylaxis, which makes an allergy to soybeans potentially life-threatening [4,5]. In adults, the soybean ranks second among the most frequent elicitors of anaphylactic reactions in the German-speaking countries [4]. A lack of therapeutic treatment to cure food allergy makes avoidance of the allergen-containing food the only option to prevent allergic reactions [3]. To support soybean allergic individuals to avoid unintended allergic reactions, labeling soybeans as an ingredient in foods is mandatory in many countries, e.g., in Europe, the USA, Canada, and Australia/New Zealand [6]. Nevertheless, allergenic soybeans may remain indiscernible or hidden due to mislabeling or the cross contact of allergen-free food products with allergenic foods, e.g., during the food manufacturing process [7]. As part of the Allergen Bureau of Australia & New Zealand, the VITAL (Voluntary Incidental Trace Allergen Labeling) expert panel established reference doses for allergenic foods on the basis of individual clinical threshold data. Accordingly, 1 mg soy protein, equivalent to 2.5 mg soy, was modeled as the eliciting dose (ED) for an allergic reaction in 5% of the soy allergic population (ED05) [8]. Thus, 95% of soy allergic subjects are thought to be safe at a level of 2.5 mg soy. Recently, the soy protein reference dose was lowered to 0.5 mg, however, without peer-reviewed publication. Accordingly, for the detection of 1.25−2.5 mg soy in a serving size of 100 g, a method sensitivity from less than 12.5 to 25 mg soy per kg food is required to verify the absence or presence of soy at a level that likely improves food safety for the majority of soy allergic subjects. The availability of specific and sensitive methods for the detection of allergenic foods is essential for the verification of compliance with labeling requirements and the risk management of cross contact with allergenic foods, such as those based on the implementation of VITAL reference doses.

Currently, the two most prominent analytical techniques for soybean detection in food are protein-based enzyme-linked immunosorbent assays (ELISA) [9,10] and nucleic acid (DNA)-based quantitative polymerase chain reaction (qPCR) assays [11,12]. Both types of methods for allergen detection have been accepted and applied by several governmental laboratories [13,14]. In addition, the detection of allergenic soybean via mass spectrometry (MS) has been reported [15,16]. However, MS, ELISA, and qPCR are procedures that require dedicated laboratory equipment and trained personnel, making them difficult or impossible for use as a simple on-site detection method in the food manufacturing environment. Here, rapid qualitative detection methods like protein-based lateral flow dipsticks (LFD) or the DNA-based loop-mediated isothermal amplification (LAMP) can provide suitable analytical tools. Although commercial protein-based methods exist for soybean detection, DNA-based analysis appears to be more robust according to a survey of proficiency testing within 6 years. Here, on average, only 5% of samples tested false-negative using DNA-based detection, while one-third of samples tested false-negative when protein-based detection, especially ELISA, was used [17]. The authors concluded that especially highly processed soy proteins were detected with low recovery rates using antibody-based ELISA tests. Accordingly, a simple DNA-based method, such as LAMP, could serve as an alternative to complement existing qPCR methods.

During the LAMP reaction, originally described by Notomi and colleagues in 2000, two or three primer pairs bind six or eight distinct regions of the target sequence, and a *Bst* polymerase with strand displacement activity initially forms a dumbbell-like structure, which then undergoes cyclic amplification, producing large amounts of various sized cauliflower-like DNA structures with alternately inverted repeats of the target sequence [18,19,20]. In contrast to PCR, the amplification reaction can be carried out under isothermal conditions (60−65 °C), eliminating the need for a thermal cycler and thus making this method highly cost-efficient and easy to perform. The only equipment required is a simple thermostatically controlled water bath, heat block, or oven [18,19].

With regard to the food and feed sector, mainly LAMP methods for the detection of foodborne pathogens [21,22] and genetically modified crops [23,24] are described. However, a number of reports suggest that LAMP may also be useful for the detection of allergenic foods [25,26,27,28]. LAMP amplicons can be detected by various methods, e.g., agarose gel electrophoresis (AGE) [25,27,28], real-time fluorescence detection using intercalating nucleic acid dyes [26,27,28], microfluidic chips for visual detection based on a pH indicator dye [26], visual detection using intercalating nucleic acid dyes [27], color changes of metal-sensitive indicators, such as calcein [28], and the visual or turbidimeter detection of turbidity formation [24]. AGE and real-time fluorescence monitoring require additional equipment for the reaction confirmation, which compromises the simplicity of LAMP and limits the field-like or on-site applications of this procedure. Visual detection of turbidity or by the use of intercalating or indicator dyes is critical in those cases where minor signal differences between positive and negative reactions are expected, for example at the limit of detection [29,30,31]. Furthermore, detection using metal-sensitive indicators, turbidity formation, and pH indicator dyes only indirectly signal a positive reaction by the detection of by-products of the LAMP-reaction, rather than LAMP amplification products [29,30,31].

Alternatively, the implementation of lateral flow devices (LFDs) as detector tools improves the visualization of LAMP products (LAMP-LFD), based on, for example, biotin and fluorescein isothiocyanate (FITC)-labeled LAMP amplicons and gold-labeled anti-FITC antibodies [32]. Here, generated gold-labeled amplicon-antibody complexes are trapped at the test line and residual unbound gold-labeled antibodies are trapped at the control line.

In addition, the amplification of multicopy genes might increase the sensitivity of DNA-based assays with special regard to a sensitive detection of allergenic foods [12,33]. However, a sensitive LAMP-LFD for the detection of allergenic soybean, which is based on multicopy gene amplification, has not been published to the best of our knowledge.

Accordingly, the present study aimed to identify suitable multicopy target genes for simple and sensitive soybean detection, based on LAMP-LFD. Target gene identification, the development and selection of specific LAMP primers sets, LFD-like amplicon detection, and the assessment of sensitivity and specificity are described in this article. Moreover, the LFD-like detection of soy-specific LAMP amplicons was compared with three other common LAMP detection methods, i.e., real-time fluorescence, AGE, and turbidity formation.

## 2. Materials and Methods 

### 2.1. Plant Material and Samples

Foods for specificity testing were purchased at local retailers or were donations by German seed breeding companies (Table 1). In addition, various soybean cultivars were analyzed (Table 2). Samples were ground using an analytical mill (M20, IKA Labortechnik, Staufen, Germany), a CryoMill (Retsch, Haan, Germany), or a knife mill (Grindomix GM200, Retsch, Haan, Germany). Finely ground flours were stored at −20 °C until further use.

### 2.2. Target Gene Selection, Alignments, and Primer Design

**Figure 1:**
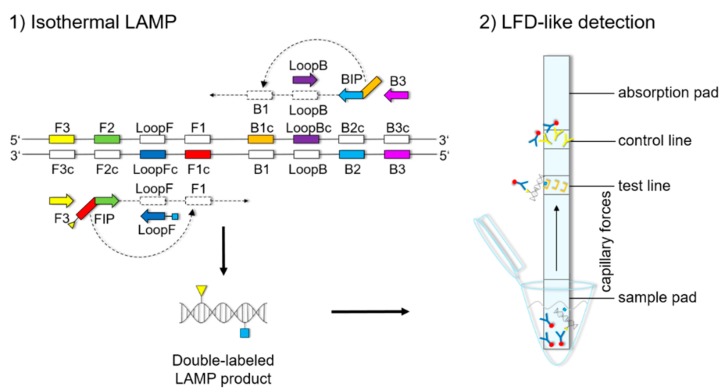
Localization of individual LAMP primers, including FITC and biotin-labeled LoopF and FIP primers, respectively, leading to a double-labeled LAMP product that is detected using a lateral flow dipstick (LFD). Illustrations of LAMP product formation are detailed elsewhere [18,19,20]. FITC- and biotin-labeled LAMP products, loaded by gold particles via an FITC-specific gold-labeled antibody, are captured on the test line by the biotin label. Free gold-labeled antibodies are captured at the control line.

Soy-specific LAMP primers were designed according to available sequences on multicopy genes for DNA detection and by screening the mitochondrial genome of soy (GenBank acc. No. JX463295.1). Various multicopy target genes were analyzed in silico for sequence fragments suitable for specific soybean DNA amplification. The *nad1*, *nad5*, *nad6*, *nad7*, *nad9*, *cox1*, *cox3*, *ORF151*, *ORF160b*, *ORF271*, *ORF287*, and *cob* gene sequences were extracted from the mitochondrial genome of soy (GenBank acc. No. JX463295.1). Additionally, the *atpA* (GenBank acc. No. Z14031.1) as well as the *ITS1* and *ITS2* (GenBank acc. No. FJ609734.1) gene sequences were analyzed. The sequence alignments (default settings) between the soybean (*Glycine max*) and other plant species were done with Vector NTI Advance^®®^ software (Version 11.5.2, Invitrogen, Carlsbad, CA, USA) and the BLAST algorithms of NCBI. Multiple alignments between the soybean (*Glycine max*) and other plant species that hold food relevance of the *atpA* sequence (Figure S1); the genes encoding ribosomal RNA and both internal transcribed spacers, *ITS1* and *ITS2* (Figure S2); and the open reading frame *ORF160b* sequence (Figure S3) were constructed to design soybean LAMP primers. The location of the individual LAMP primers is depicted in principle in Figure 1, and in detail in Figures S1–S3 (supplementary material). LAMP primers were designed either by using the online software Primer Explorer v5 or manually, verifying the primer characteristics with the online NetPrimer Software [34,35]. If possible, primer design followed the instructions on the Eiken Genome Site [36]. To assess the in silico specificity of the newly designed primers, a search for homologous sequences was performed using BLAST at the NCBI homepage. For LFD detection of the *atpA* and the *ORF160b*-specific sequences, the corresponding primers FIP and LoopF were labeled with 5′-biotin and 5′-FITC, respectively. All oligonucleotides were commercially synthesized (biomers.net GmbH, Ulm, Germany). Details about the LAMP primer sequences are displayed in Table 3.

### 2.3. DNA Extraction and Purification

The DNA extraction was performed as previously described, with some modification [37,38]. Briefly, 100 mg of ground sample, 1.4 mL of cetyltrimethylammonium bromide (CTAB) buffer (55 mM CTAB, 1400 mM NaCl, 20 mM ethylenediaminetetraacetic acid (EDTA)·2Na^+^ ·2H_2_O, 100 mM tris(hydroxymethyl)aminomethane (Tris)), pH 8.0, and 20 μL of proteinase K were mixed in a 2 mL micro reaction tube and incubated at 65 °C for 60 min and 1000 rpm in a “Thermomixer comfort” (Eppendorf, Hamburg, Germany). After centrifugation at 13,200 rpm for 10 min, 800 μL of supernatant was transferred to a 2 mL micro reaction tube and vortexed with 600 μL of chloroform. After centrifugation for 5 min, 600 μL of the supernatant was added to 1 μL of mussel glycogen in another 2 mL micro reaction tube, and after short mixing, 500 μL of chilled (−20 °C) isopropanol was added. The tube was carefully inverted 10 times and centrifuged. After supernatant removal, the DNA pellet was washed with 500 μL of 70% ethanol and briefly centrifuged. After removal of ethanol, the pellet was dried at 50 °C for 2−5 min. Afterwards, the dried DNA pellet was resuspended and resolved in 100 μL of TE buffer (10 mM Tris HCl, 1 mM EDTA Na_2_·2H_2_O), pH 8.0, overnight at 4 °C, and 300 rpm using the Thermomixer comfort. The resuspended DNA pellet was further purified using silica spin columns by following the manual of the QIAquick PCR Purification Kit (Qiagen, Hilden, Germany). The DNA was eluted twice with 50 μL of EB buffer (Qiagen). The DNA extracts were stored at −20 °C until use. UV spectrometric DNA quantification was performed with an Implen NP80 NanoPhotometer (Munich, Germany). The DNA concentration was measured based on UV absorption at 260 nm (1 absorbance unit corresponds to 50 ng/μL of dsDNA), while DNA purity was evaluated based on the UV absorption value ratio at 260/280 nm. 

### 2.4. Real-time PCR for the Detection of Eukaryotic DNA

To exclude the presence of inhibitors that possibly lead to false negative results, the quality of DNA extracts was confirmed by amplification with the previously published universal eukaryotic PCR primers TR03/TR04 (Table 3) [39]. The binding region for this universal primer set is located in the 18S coding region of the rDNA showing high sequence homology for eukaryotic species. Amplification reactions were monitored by real-time fluorescence detection using an MX 3005P™ real-time PCR detection system (Stratagene, La Jolla, CA, USA). The composition of the Mastermix for eukaryotic real-time PCR was 1× Taq DNA Polymerase PCR Buffer (Invitrogen, Life Technologies, Carlsbad, CA, USA); 200 μM each of dATP, dCTP, and dGTP; 400 µM dUTP (Carl Roth, Karlsruhe, Germany); 2 mM MgCl_2_ (Fisher Scientific GmbH, Schwerte, Germany); 2 µg/mL bovine serum albumin (BSA) solution (Sigma Aldrich, St. Louis, USA); 0.5 µM of each sense and antisense primer; 1:60,000 diluted SYBR Green I Nucleic Acid Gel Stain (Roche, Mannheim, Germany); 0.01 Units/μL Uracil-N-glycosylase (UNG, Jena Bioscience, Jena, Germany); 0.025 Units/μL Platinum Taq DNA polymerase (Invitrogen, Life Technologies); and 5 μL of 1:10 diluted sample DNA and sterile water that was added to a final volume of 25 μL. The eukaryotic real-time PCR program consisted of a first UNG digestion step at 50 °C for 2 min, followed by deactivation of the UNG and initial denaturation for 10 min at 95 °C, and 40 cycles each of 30 s at 94 °C for denaturation, 30 s at 60 °C for primer annealing, and 1 min at 72 °C for elongation. 

### 2.5. Basic LAMP Protocol

LAMP reactions for initial screening of sensitivity and specificity of various target genes were carried out in a total reaction volume of 25 μL containing 0.2 μM of each F3 and B3, 1.6 μM of each FIP and BIP, 0.4 μM of each LoopF and LoopB, 1.4 mM of each dNTP (Carl Roth), 1.4 M dimethyl sulfoxide (DMSO; Carl Roth), 8 U *Bst* 2.0 WarmStart^®®^ DNA Polymerase (New England BioLabs, Frankfurt am Main, Germany), 20 mM Tris-HCl (pH 8.8), 10 mM (NH_4_)2SO_4_, 50 mM KCl, 8 mM MgSO_4_, 0.1% Tween^®®^ 20, 1:60,000 diluted SYBR Green I Nucleic Acid Gel Stain (Roche), and 5 μL of template DNA. The reaction mixture was incubated for 60 min at 61−65 °C, depending on the primer set used (Table 3). For no template controls (NTCs), LAMP mixtures contained 5 μL of sterile water instead of DNA template.

### 2.6. Final atpA LAMP Protocol

After optimization, LAMP reactions targeting the *atpA* gene were carried out in a total volume of 25 μL containing 0.2 μM of each F3 and B3, 0.8 μM of each FIP and Biotin-FIP, 1.6 µM BIP, 0.1 μM FITC-LoopF, 0.3 µM LoopF, 0.4 µM LoopB, 1.4 mM of each dNTP (Carl Roth), 1.4 M DMSO (Carl Roth), 8 U *Bst* 2.0 WarmStart^®®^ DNA Polymerase (New England BioLabs), 20 mM Tris-HCl (pH 8.8), 10 mM (NH_4_)2SO_4_, 50 mM KCl, 8 mM MgSO_4_, 0.1% Tween^®®^ 20, 1:60,000 diluted SYBR Green I Nucleic Acid Gel Stain (Roche), and 5 μL of template DNA. Reactions were incubated at 63 °C for 35 min.

### 2.7. Final ORF160b LAMP Protocol

After optimization, LAMP reactions targeting the *ORF160b* were carried out in a total reaction volume of 25 μL containing 0.15 μM of each F3 and B3, 0.6 μM of each FIP and Biotin-FIP, 1.2 µM BIP, 0.075 μM FITC-LoopF, 0.225 µM LoopF, 0.3 µM LoopB, 1.4 mM of each dNTP (Carl Roth), 0.8 M DMSO (Carl Roth), 0.25 mg/mL BSA solution (Sigma Aldrich), 8 U Bst 2.0 WarmStart^®®^ DNA Polymerase (New England BioLabs), 20 mM Tris-HCl (pH 8.8), 10 mM (NH_4_)2SO_4_, 50 mM KCl, 6 mM MgSO_4_, 0.1% Tween^®®^ 20, 1:60,000 diluted SYBR Green I Nucleic Acid Gel Stain (Roche), and 5 μL of template DNA. Reactions were incubated for 50 min at 62 °C. 

### 2.8. LAMP Detection Methods

During the development process, amplification reactions were monitored by real-time fluorescence detection, AGE, turbidity formation, and LFD-like detection.

The LFD-like detection was carried out using commercially available dipsticks (Milenia HybriDetect, Milenia Biotec GmbH, Gießen, Germany). LAMP experiments were performed as described above, using a biotinylated FIP primer and an FITC-labeled LoopF primer, resulting in a double-labeled LAMP product (Figure 1). Two microliters of LAMP product were mixed with 100 μL of 1% TBST buffer (50 mM Tris, 150 mM NaCl, 34.6 mM HCl 37%, 1% Tween20^®®^, pH 7.4) in a new tube. The LFD was dipped into the mixture for 5 min. The double-labeled LAMP product is bound to a gold-labeled rabbit-anti-FITC-antibody that has been preloaded on the sample pad. Driven by capillary forces, the sample fluid flows through the membrane up to the test line where the double-labeled LAMP product is captured via the biotin label. Only the double-labeled LAMP product/gold-labeled antibody complex will then generate a colored band signaling a positive reaction. Excess of gold-labeled rabbit anti-FITC antibodies will flow up to the control line and be bound by anti-rabbit antibodies, signaling optimal reaction conditions. For a negative sample, only the control line will be visible.

Monitoring of fluorescence in real-time was done using an MX 3005P™ real-time PCR detection system (Stratagene) or a StepOnePlus real-time PCR system (Applied Biosystems, Foster City, CA, USA). At every minute of amplification, the increase of fluorescence intensity of SYBR Green I that is intercalated into the amplified DNA was measured using the carboxyfluorescein (FAM)-channel (excitation and emission 492/517 nm). The data generated with SYBR Green I were plotted as the relative fluorescence signal versus time (each cycle was set to 1 min at a constant temperature). The threshold cycle (Ct) used for real-time PCR assays was converted into threshold time (T_T_), i.e., the time (min) required for the fluorescence signal to cross an arbitrary fluorescence threshold (F_T_) [28,32]. 

AGE was done with the “Rapid Agarose Gel Electrophoresis System” (RAGE system, Cascade Biologics, Portland, OR, USA). Post-LAMP, 6 μL of LAMP product were mixed with 6× loading buffer and loaded onto a 3% (*w*/*v*) high-resolution agarose gel (Carl Roth). The sizes of the LAMP products were analyzed by comparison with the “O’Gene Ruler 1kb Plus DNA Ladder” (Fermentas, Waltham, USA) in gel electrophoresis with 1× TAE buffer (40 mM Tris, 20 mM acetic acid and 1 mM EDTA), pH 8.0, at 150 V (25 mL gel) for approximately 15−20 min. After gel electrophoresis, the gel was stained in 0.75 μg/mL ethidium bromide (Carl Roth) for 15 min. The results were visualized and digitalized under UV light on a UV (312 nm) transilluminator (Intas Gel Jet Imager, Intas Science Imaging Instruments GmbH, Göttingen, Germany).

### 2.9. Assessment of Primer Sensitivity and Specificity

To estimate the sensitivity of the LAMP assays, DNA was extracted from ground yellow soybeans (Schoenenberger^®®^ Hensel^®®^, Magstadt, Germany), and serial DNA dilutions between 1:10^3^ and 1:10^7^ (simulating amounts between 1000 and 0.1 mg soybean per kg sample) were prepared as templates. Each dilution level was analyzed in duplicate. LAMP reactions were performed according to the above-described basic LAMP protocol. In NTCs, DNA template was substituted with sterile water. The LAMP reactions were analyzed by real-time fluorescence monitoring as described above. The lowest level of detectability for each primer set was defined as the lowest level of soy DNA dilution at which amplification occurred for all investigated replicates.

An initial assessment of primer specificity was done especially with members of the *Fabaceae* family that represent relevant potential cross-reactive food components, i.e., peanuts; chickpeas; kidney and pinto beans; brown and red lentils; peas; and blue, yellow, and white lupines. In addition, barley and wheat were tested as representatives of commonly used food ingredients (Table 1). Extracted genomic DNA was analyzed undiluted and diluted 1:100 in sterile water. DNA extracted from (soybean) tofu served as a positive control. LAMP reactions were performed according to the above-described basic LAMP protocol. The LAMP reactions were analyzed by real-time fluorescence monitoring and AGE as described above.

After selection and optimization of the most specific gene/primer candidates, extensive specificity testing of the *atpA* and the *ORF160b* LAMP assays was performed using genomic DNA from 33 species (Table 1). Especially members of the *Fabaceae* family and other common ingredients that hold relevance because of mandatory ingredient labeling according to European legislation [40] were selected. DNA was tested undiluted and diluted 1:100 in sterile water. DNA extracted from tofu served as a positive control. *atpA* and *ORF160b* LAMP reactions were carried out as described above. Within 35 min of incubation in the case of the *atpA* LAMP assay and 50 min incubation in the case of the *ORF160b* LAMP assay, the reactions were monitored by real-time fluorescence. Endpoint reactions were analyzed with AGE and LFD as described above.

### 2.10. Analyses of Soybean Cultivars

DNA was extracted from seeds of 11 different soybean cultivars (Table 2). Dilutions of soybean DNA in sterile water were prepared between 1:10^3^ and 1:10^7^ (simulating amounts of 0.1–1000 mg soybean per kg food) as templates for the LAMP-LFD reactions. Each level of dilution was analyzed in duplicate. LAMP-LFD reactions were performed as described above. DNA from ground yellow soybeans (Schoenenberger^®®^ Hensel^®®^) served as a positive control. 

## 3. Results and Discussion

### 3.1. Target Gene Selection, Alignments, and Primer Design

For gene target selection with special regard to the isothermal LAMP detection of soybeans, several considerations had to be taken into account. The target gene must provide sequence variation compared to other species that are relevant for food ingredients. The sequence stretch requires a certain length of approximately 200−500 bp to accommodate all LAMP primers. Similar to PCR, the LAMP target sequence should be as short as possible, since processing during food manufacture could lead to sequence fragmentation. Various multicopy target genes were evaluated in this regard. Multiple sequence alignments resulted in insufficient variation at the site of the *nad1*, *nad5*, *nad6*, *nad7*, *nad9*, *cox1*, *cox3*, *ORF151*, *ORF271*, *ORF287*, and *cob* sequences between soybean and the other plant species. In silico analysis suggested *atpA* (GenBank acc. No. Z14031.1), *ITS* (GenBank acc. No. FJ609734.1), and *ORF160b* (GenBank acc. No. JX463295.1) as promising target genes. The use of the mitochondrial *atpA* gene and the *ITS* sequences of 18S-26S nuclear rDNA, as targets, have formerly been reported for an increase in sensitivity, compared to single copy DNA targets [12,33]. According to Chang and colleagues (2013), predicted open reading frames (ORFs) of undefined function in mitochondrial genomes vary more than the genes of known function among plant species within a closely related clade and thus might be feasible targets for specific soybean detection [41]. 

From the alignment results, five LAMP primer sets for the detection of soybean were designed on the basis of the interspecies sequence variation of the soybean *atpA* (GenBank acc. No. Z14031.1), *ITS1* (GenBank acc. No. FJ609734.1), and *ITS2* (GenBank acc. No. FJ609734.1) genes and the *ORF160b* (GenBank acc. No. JX463295.1) (Table 3; Figures S1–S3). The orientation and positions of the interrelated set of primers are important for self-priming through stem-looped products that drive the reaction. The LAMP method is based on a set of four specially designed primers (F3, B3, FIP, and BIP) that recognize six distinct fragments (F1, F2, F3, B1, B2, and B3) of the target sequence (Figure 1). The two inner primers, FIP and BIP, consist of two distinct sequences targeting two different sequence fragments on both single strands of the double-stranded DNA molecule. FIP consists of complementary sequence F1 (F1c) and F2. BIP consists of complementary sequence B1 (B1c) and B2. The two outer primers, F3 and B3c, are located outside the F2-B2 region [42]. To increase amplification efficacy, two loop primers, the forward loop primer (LoopF) and backward loop primer (LoopB), were designed. The sequence alignments and directions of primers are shown in Figures S1–S3. In some references, a spacer was inserted between F1c or B1c and F2 or B2 in the inner primer (FIP or BIP) [16,39]. However, the spacer was not used in this study, because it was shown that the LAMP reaction can also progress without the use of spacers [25,27]. Primer sets were designed to target the *atpA*, *ITS1*, *ITS2*, and the *ITS1* and *ITS2* gene spanning the whole 5.8S gene and to target the *ORF160b* (Table 3, Figures S1–S3). For subsequent LFD detection, primers FIP and LoopF of the *atpA* and the *ORF160b* primer sets were additionally 5′-biotin-labeled and 5′-FITC-labeled, respectively.

Except for the *atpA* primer set, which shows homologies with the broad bean (*Vicia faba*), all of the newly designed primers lack relevant homologies with other closely-related species, as investigated in silico by NCBI BLAST search.

### 3.2. Pre-Selection of Primer Sets According to Sensitivity and Specificity 

To ensure that the majority of soybean allergic consumers are appropriately protected, we aimed to detect soybean at a level at or below 12.5−25 mg soybean per kg of food. Dilution series of target DNA between 1:10^3^ and 1:10^7^ were used to simulate soybean levels between 0.1 and 1000 mg soybean per kg of food (Table 4).

Initial experiments with serially diluted DNA indicated appropriate sensitivity of the *atpA*, the *ITS2*, and the *ORF160b* LAMP assays (Table 4), while the negative controls (NTC), having no DNA template, remained negative. The successful amplification of a 1:10^5^ dilution simulated a potential detectability of 10 mg soybean per kg food. The *ITS1* primer set could not provide the needed sensitivity of 10 mg soybean per kg food and led to false positive test results. The *ITS1 + 2* primer set continuously generated false positive test results for the NTC. Hence, both the *ITS1* and the *ITS1 + 2* primer sets were excluded from further evaluation.

Subsequently, the specificity of the preselected primer sets for *atpA*, *ITS2*, and *ORF160b* was investigated experimentally. To verify the in silico findings, especially legume species were selected for an initial specificity assessment (Table 5). Genomic DNA from these 12 food samples was investigated undiluted and 1:100 diluted in the three LAMP assays and a eukaryotic 18S rRNA control assay. The successful amplification of a conserved eukaryotic sequence on the 18S rRNA gene indicated the absence of PCR inhibitors and the integrity of the extracted DNA, respectively.

In addition to soy (tofu) DNA, the *ITS2* LAMP assay also amplified DNA from peanuts, barley, wheat, red lentils, and blue lupines, indicating that the *ITS2* primers are cross-reacting with different species of various plant families. Hence, the *ITS2* LAMP assay was excluded from further experiments.

In addition to soy (tofu) DNA, the *atpA* LAMP assays also amplified DNA from legumes, including chickpeas, kidney and pinto beans, brown and red lentils, and peas, indicating that the *atpA* primers are cross-reacting with additional members of the *Fabaceae* family. By contrast, peanut and lupine DNA were not amplified. No cross-reactions were observed with DNA from the other tested plant family members. Consequently, these primers may not be defined as “specific” for soybeans, but likely as “selective” for some members of the *Fabaceae* family, excluding peanuts and lupines.

Finally, the *ORF160b* LAMP assay amplified only soy (tofu) DNA and thus indicated the highest specificity among the investigated LAMP assays (Table 5). Both the *ORF160b* and *atpA* LAMP assays were further optimized and investigated in detail.

### 3.3. Primer Labeling and Assay Optimization

Starting with the basic LAMP protocol, both the *atpA* and *ORF160b* LAMP assays were optimized to achieve detectable amplification within a possibly short reaction time. First, in order to allow for an LFD-like detection of amplified DNA, LAMP primers FIP and LoopF were partially biotinylated and FITC-labeled, respectively. The ratio of labeled primers (Table 6) was optimized to allow for both fluorescence-based real-time monitoring and later LFD-like detection. 

**Figure 2:**
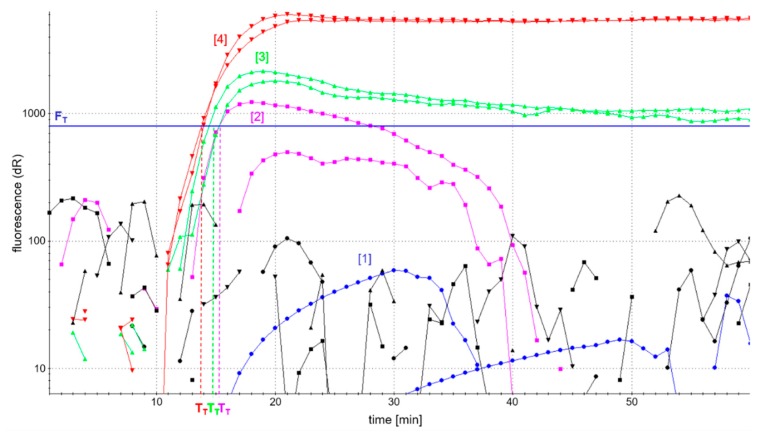
Optimization of the ratio of labeled biotin-FIP and FITC-LoopF primers in fluorescence-based real-time detection of the *atpA* gene. FIP/biotin-FIP and LoopF/FITC-LoopF primer ratios: blue [1] = 0:1, 0:1; magenta [2] = 0:1, 1:1; green [3] = 1:1, 1:1; red [4] = 1:1; 1:3; black = reaction without DNA template (NTC).

LAMP reactions, according to the basic LAMP protocol, were carried out using 1:10^3^ diluted soy DNA, and effects on reaction performance were monitored in real-time based on fluorescence development of the DNA intercalating dye SYBR Green I. Figure 2 shows the fluorescence-based detection of the *atpA* LAMP as an example. In addition, patterns of amplification products were analyzed in AGE to control for comparable amplification in the case of low or fully quenched fluorescence. Efficient accumulation of amplification products was evaluated by a possibly high fluorescence development and short reaction time to reach fluorescence maxima in real-time detection, as well as a comparable pattern of amplification products as assessed by AGE. 

**Figure 3:**
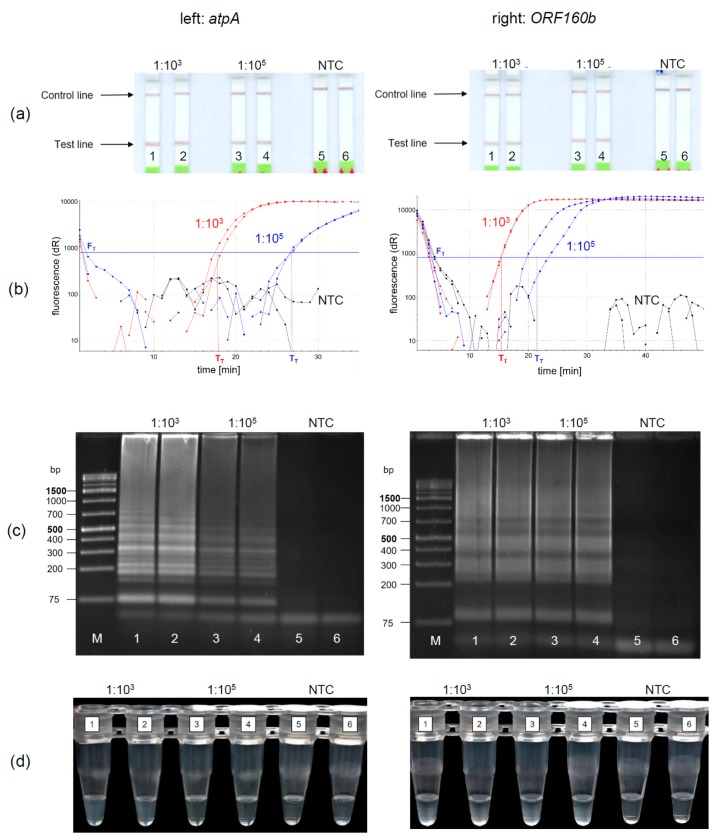
Duplicate detection of LAMP DNA products of the optimized *atpA* (left) and *ORF160b* (right) LAMP assays using (**a**) the lateral flow dipstick (LFD); (**b**) real-time fluorescence based on SYBR Green I; (**c**) agarose gel electrophoresis (AGE); (**d**) visual inspection via turbidity formation. (**b**: red curves: 1:10^3^ diluted DNA; blue curves: 1:10^5^ diluted DNA; black curves: NTC).

The analysis of LAMP amplicons using AGE showed successful amplification patterns, comparable to those depicted in Figure 3c, and independent of the four different primer ratios investigated. However, the development of fluorescence was dependent on the concentration of labeled primers (Figure 2). The use of fully labeled primers (blue curve) completely quenched fluorescence. By contrast, the fluorescence intensity of *atpA* LAMP reactions increased with decreasing concentrations of labeled primers. The onset of the detectable fluorescence increase (dR) of *atpA* LAMP reactions with all combinations of partially labeled primers started almost simultaneously after around 15 min of incubation time. In all further experiments, an FIP/biotin-FIP primer ratio of 1:1 and LoopF/FITC-LoopF primer ratio of 1:3 (Figure 2, red curve) was used, since it enabled the highest fluorescence development in real-time monitoring of all investigated ratios of primer labeling. 

Furthermore, the reaction conditions of relevant parameters were investigated in detail: reaction temperature as well as the concentration of the total primer, *Bst* polymerase, MgSO_4_, and potential reaction enhancers DMSO and betaine (Table 7). Again, 1:10^3^ diluted soybean DNA was investigated in all varied reaction conditions according to Table 7. 

Regarding the *atpA* LAMP assay, the composition of the basic LAMP reaction mixture already reflected optimal reaction conditions. Only the reaction temperature had an additional impact on assay time. Detectable LAMP products were generated at a temperature range of 60−66 °C, with a temperature optimum of 63 °C leading to the shortest assay time of 15 ± 1 min according to fluorescence emission above F_T_.

By contrast, all analyzed parameters of the *ORF160b* LAMP assay, except for the polymerase concentration, had an impact on assay time. The decrease of Mg^2+^ concentration from 8 to 6 mM had the highest impact on reaction time and allowed fluorescence-based detection around 17 ± 2 min instead of 37 min before optimization. For both LAMP assays, the optimized conditions are summarized in Table 7 and the Materials and Methods section. These reaction conditions were used in all further experiments.

### 3.4. Evaluation of Detection Methods for LAMP Amplification Products

Under the optimized reaction conditions of the soybean *atpA* and the *ORF160b* loop-mediated isothermal DNA amplification, four different methods for the detection of amplified soybean DNA were evaluated. The LFD-like detection of LAMP products was compared with real-time fluorescence detection, AGE, and turbidity formation. Soybean DNA was diluted 1:10^3^ and 1:10^5^ to simulate equivalent amounts of 10 and 1000 mg soybean per kg food and amplified and detected in duplicates. Using the LFD-like detection, a positive sample is visualized by two lines, the test and the control line, while for negative samples only the control line would be visible. LFD-like detection of amplification products of both the *atpA* (Figure 3a, left) and *ORF160b* (Figure 3a, right) assay resulted in clearly visible reddish bands at the test lines for the 1:10^3^ and 1:10^5^ diluted soybean DNA. It should be noted that the color intensity of the test line was independent of the initial soybean concentration tested. No amplification was detected in the negative controls (NTC).

Using real-time fluorescence monitoring (Figure 3b), both levels of diluted soybean DNA were clearly detectable in both LAMP assays. Diluted soybean DNA with ratios of 1:10^3^/1:10^5^ was detectable within 15 min/22 min and 18 min/27 min using the *atpA* and *ORF160b* LAMP assay, respectively. Accumulation of *ORF160b* LAMP products was saturated after 20−30 min compared to approximately 25−35 min using the *atpA* LAMP assay. Fluorescence did not exceed the threshold signal F_T_ in the negative controls (NTC).

Detection of LAMP products using AGE is shown in Figure 3c. Typical ladder-like bands of various-sized amplification products are formed and detected. No amplification occurred in the negative controls (NTC). Turbidity formation (Figure 3d) is based on the production of insoluble magnesium pyrophosphate as a by-product in the course of the LAMP amplification reaction [43].

This precipitate might become visible, after the initially clear solution turns cloudy during the course of amplification.

Using *atpA* (left) as a target sequence, only 1:10^3^ diluted soy DNA resulted in visually detectable precipitates (Figure 3d left, tubes 1, 2). Diluted soy DNA with a ratio of 1:10^5^ and the NTCs did not form visible precipitates. In comparison, precipitates resulting from the *ORF160b* LAMP (right) were visible in all samples having soy DNA (Figure 3d right, Tubes 1−4), while the NTCs showed no precipitate (Tubes 5 and 6). When comparing visual turbidity with any other type of detection, the results were difficult to interpret and partially inconsistent. Hence, subjective turbidity was not a preferred detection method in our hands. LAMP detection using AGE or real-time fluorescence was as reproducible and clear as LFD-like detection but required additional laboratory equipment. By contrast, the LFD-like detection of LAMP amplicons allowed for clear visual detection and was easy and rapid to perform without the need of additional equipment. Moreover, the color intensity of the test line was independent of the initial concentration of the samples tested. Thus, even low levels of soybean DNA resulted in a clearly visible positive test line. By contrast, protein-based LFD-like detection, using antibodies, usually results in weak positive test lines at low antigen concentrations [44]. However, in the field of allergen detection, especially low levels of the respective allergenic food should be clearly detectable.

### 3.5. Extensive Specificity Testing of atpA and ORF160b LAMP-LFD Assays

**Figure 4:**
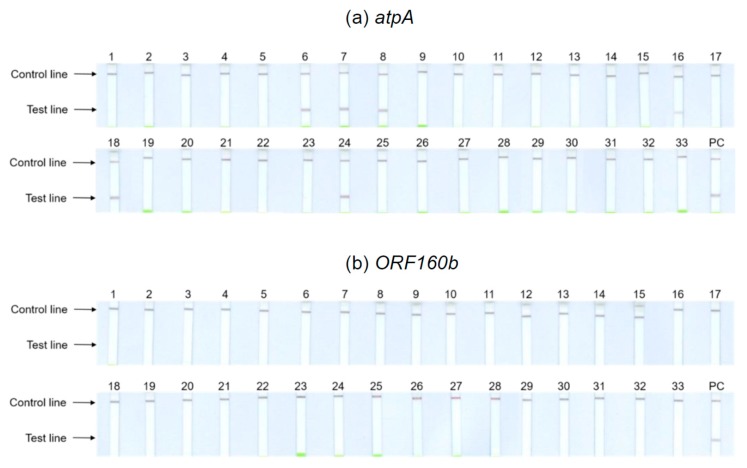
Specificity of (**a**) the *atpA* and (**b**) the *ORF160b* LAMP-LFD assays for the detection of allergenic soybean. Cross-reactivity testing with undiluted DNA. Numbers according to Table 1 (PC: positive control tofu).

Thirty-three different kinds of foods purchased in local supermarkets and received from seed breeding companies were included in specificity testing of the *atpA* and *ORF160b* LAMP-LFD assays (Table 1), including those foods that require mandatory labeling when used as ingredients according to EU legislation 1169/2011 [40]. Food samples were tested for cross-reactivity on the basis of undiluted and 1:100 diluted DNA extracts. Figure 4 depicts the results obtained with the undiluted DNA of potential cross-reactants. Tests with 1:100 dilutions showed identical results. The *atpA* LAMP-LFD assay (Figure 4a) cross-reacted with the legumes including chickpeas (6), kidney (7) and pinto (8) beans, brown (16) and red (24) lentils, and peas (18). With the remaining tested foods, no further cross-reactivities could be observed. Hence, the results of the initial specificity screening were confirmed. By contrast, the *ORF160b* LAMP-LFD assay (Figure 4b) was highly specific for soy and did not cross-detect any of the other tested species.

The obtained LAMP amplification products were also visualized using real-time fluorescence detection and AGE, and the results of both alternative detection methods were consistent with those obtained with the LAMP-LFD detection system.

### 3.6. Analyses of Soybean Cultivars

**Figure 5:**
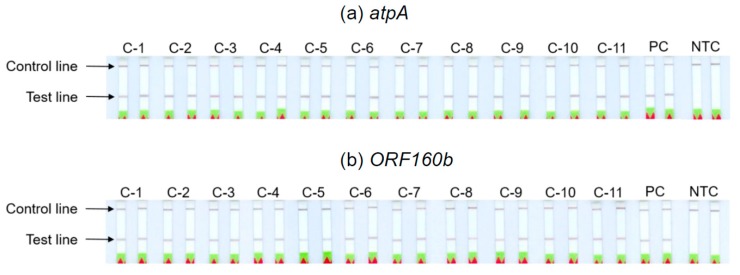
Screening of different soybean cultivars using (**a**) the *atpA* and (**b**) the *ORF160b* LAMP-LFD assays. Duplicate analysis of 1:10^5^ diluted DNA (equivalent to 10 mg soybean per kg food) of the 11 soybean cultivars C-1 to C-11 according to Table 2 (PC: positive control yellow soybean (Schoenenberger^®®^ Hensel^®®^); NTC: no template controls).

The ability of the *atpA* and *ORF160b* LAMP-LFD assays to detect different soybean cultivars was investigated (Table 2). Serial dilutions of DNA extracted from seeds of 11 different soybean cultivars with ratios of 1:10^3^ to 1:10^7^ were prepared as templates for the LAMP-LFD reactions, in order to simulate amounts equivalent to 0.1−1000 mg soybean per kg food. The successful duplicate amplification and detection of 1:10^5^ diluted soybean DNA indicated a sensitivity to potentially detect 10 mg of soybean per kg food, independent of the tested cultivars (Table 2, Figure 5). The sensitivity of both LAMP-LFD assays to detect various soybean cultivars was comparable.

## 4. Conclusions

This study provides a proof of concept for multicopy gene targets that can be used in LAMP assays to allow for a sensitive, specific, and simple detection of allergenic soybean. Overall, the two identified gene targets, *atpA* and *ORF160b*, provided a comparably sensitive detection of allergenic soybean using LAMP combined with LFD detection. In addition, the *ORF160b* LAMP-LFD assay proved to be highly specific for soybeans, with no other cross-reactivity observed in this study. Moreover, the *ORF160b* primers allowed for an even faster amplification of soybean DNA than the *atpA* specific primers. The LFD-like visualization of LAMP amplicons allowed for a simple and unambiguous detection, which fully complied with the results obtained by real-time fluorescence monitoring and AGE, respectively. Further, LFD-like detection of LAMP amplicons did not require additional or expensive laboratory equipment. These features are beneficial for use in field-like environments or on-site applications, such as the manufacturing of foods. Independently, such simple analytical tools for allergen detection are potentially useful for a primary screening in any laboratory. The DNA extraction protocol used in this study was adopted from our previous work on the PCR detection of allergenic foods [37,38]. There, it allowed for an efficient purification of DNA from a range of complex food matrixes, such as chocolate, cookies, cereals, muesli, and ice cream, in order to verify the presence of allergens, e.g., hazelnuts or peanuts, at or below a level of 10 mg per kg food using qPCR. Using this established DNA extraction protocol, isolates may be used for a LAMP-LFD screening, followed by verification with established qPCR methods, if verification or quantitative results are needed. The DNA extraction protocol used in this study is still a lengthy procedure. If combined with a rapid DNA extraction protocol, the presented LAMP-LFD has potential as a rapid method for on-site applications.

This study focused on the development of specific and sensitive primers for a simple and ideally rapid amplification and detection of DNA from allergenic soybean, complementary to existing real-time PCR methodology. Especially the *ORF160b* LAMP primers showed high potential for specific and sensitive soybean detection by a simple means. Except for tofu made from soybean, none of the foods used in this study, either as a reference or as a sample, had undergone thermal processing. As a next step, to elucidate the full potential of the identified LAMP primers for the detection of allergenic soybean in foods, the impact of thermal processing on detectability; additional method validation in various different food matrices, such as the determination of the limit of detection; and performance testing versus existing protein-based and DNA-based soybean detection methods will follow.

## Figures and Tables

**Table 1:** Food samples used in this study.

Sample No.	Common Name	Species Name	Family
1	almond ^1^	*Prunus dulcis*	*Rosaceae*
2	pistachio ^1^	*Pistacia vera*	*Anacardiaceae*
3	cashew ^1^	*Anacardium occidentale*	*Anacardiaceae*
4	pecan ^1^	*Carya illinoinensis*	*Juglandaceae*
5	peanut ^1,^ ^2^	*Arachis hypogaea*	*Fabaceae*
6	chickpea ^2^	*Cicer arietinum*	*Fabaceae*
7	kidney bean ^2^	*Phaseolus vulgaris*	*Fabaceae*
8	pinto bean ^2^	*Phaseolus vulgaris*	*Fabaceae*
9	sesame seed ^1^	*Sesamum indicum*	*Pedaliaceae*
10	cow’s milk ^1^	*Bos taurus*	*Bovidae*
11	canned skipjack tuna ^1^	*Katsuwonus pelamis*	*Scombridae*
12	oat ^1^	*Avena sativa*	*Poaceae*
13	barley ^1^	*Hordeum vulgare*	*Poaceae*
14	rye ^1^	*Secale cereale*	*Poaceae*
15	wheat ^1^	*Triticum sp.*	*Poaceae*
16	brown lentil ^2^	*Lens culinaris*	*Fabaceae*
17	walnut ^1^	*Juglans regia*	*Juglandaceae*
18	pea ^2^	*Pisum sativum*	*Fabaceae*
19	brazil nut ^1^	*Bertholletia excelsa*	*Lecythidaceae*
20	macadamia ^1^	*Macadamia sp.*	*Proteaceae*
21	white mustard seed ^1^	*Sinapis alba*	*Brassicaceae*
22	white shrimp ^1^	*Litopenaeus vannamei*	*Penaeidae*
23	hen’s egg ^1^	*Gallus gallus domesticus*	*Phasianidae*
24	red lentil ^2^	*Lens culinaris*	*Fabaceae*
25	buckwheat	*Fagopyrum esculentum*	*Polygonaceae*
26	blue lupine ^1, 2^	*Lupinus angustifolius*	*Fabaceae*
27	yellow lupine ^1, 2^	*Lupinus luteus*	*Fabaceae*
28	white lupine ^1, 2^	*Lupinus albus*	*Fabaceae*
29	hazelnut ^1^	*Corylus avellana*	*Betulaceae*
30	spelt ^1^	*Triticum aestivum* subsp. *spelta*	*Poaceae*
31	squid ^1^	*Loligo formosana*	*Loliginidae*
32	blue mussel ^1^	*Mytilus edulis*	*Mytilidae*
33	celeriac ^1^	*Apium graveolens* var. *rapaceum*	*Apiaceae*
34	100% tofu ^1, 2^	*Glycine max*	*Fabaceae*
35	soybean ^1, 2^	*Glycine max*	*Fabaceae*

^1^ requires mandatory labeling as an ingredient according to EU Directive 1169/2011; ^2^ legume (*Fabaceae*).

**Table 2:** Screening of different soybean cultivars using both LAMP-LFD assays based on diluted soybean DNA. (+: 2/2 replicates positive; (+): 1/2 replicates positive; -: 2/2 replicates negative).

Sample No.	Cultivar	*atpA* LAMP-LFD				*ORF160b* LAMP-LFD			
		Diluted Soybean DNA							
		1:10^3^	1:10^5^	1:10^6^	1:10^7^	1:10^3^	1:10^5^	1:10^6^	1:10^7^
C-1	Merlin ^1^	+	+	-	-	+	+	-	-
C-2	Lissabon ^1^	+	+	-	-	+	+	-	-
C-3	Malaga ^1^	+	+	(+)	-	+	+	-	-
C-4	Amarok ^2^	+	+	-	-	+	+	-	-
C-5	Pollux ^2^	+	+	-	-	+	+	-	-
C-6	Tiguan ^3^	+	+	(+)	-	+	+	-	-
C-7	Falbala ^3^	+	+	-	-	+	+	-	-
C-8	Orion ^3^	+	+	-	-	+	+	(+)	-
C-9	Idefix ^3^	+	+	-	-	+	+	-	-
C-10	Obelix ^3^	+	+	-	-	+	+	(+)	-
C-11	Toutatis ^3^	+	+	-	-	+	+	-	-
35	yellow soybeans (Schoenenberger^®®^ Hensel^®®^)	+	+	(+)	-	+	+	(+)	-

^1^ seed dealer: Saatbau Linz eGen, Austria; ^2^ seed dealer: BayWa Gruendl, Germany; ^3^ seed dealer: Delley Seeds and Plants Ltd., Switzerland.

**Table 3:** LAMP primers and 18S-eukaryote PCR primers used in this study (except for 18S rRNA, all primers were developed in this study).

Target Gene (T_A_) ^1^	Primer Name	Sequence (5′→3′)
*atpA* (63 °C)	F3	GATATCTTGGTTTTGGAAAACTG
	B3	GCTATGTTCTGAGATCCGTC
	FIP	TAGCCCAGGCTAACTAGAATAGTTATTAAAAAGACGAATGCTCCTC
	biotin-FIP	b-TAGCCCAGGCTAACTAGAATAGTTATTAAAAAGACGAATGCTCCTC
	BIP	AGAAGACCCTGTCAAGCCTTTGTGCTGTTCAAAGATCTCTCC
	LoopF	TCCCAAAGCAATAACCACAGATG
	FITC-LoopF	FITC-TCCCAAAGCAATAACCACAGATG
	LoopB	GGGCAATGCCACTTCGAAAG
*ITS1* (64 °C)	F3	TGCCTCACAATCAGATTGACC
	B3	AAATGTTGTGTCGTGACGACTC
	FIP	GGGGTTTGTGTTTGTCGCGAGGGAGGGGATGACCAC
	BIP	CGCTTCGTGCGCCAAGGCGGGACACCGTCTCC
	LoopF	GGACGAGGAGGCCGGG
	LoopB	CGAAATCTGTTAAGTGCGACTC
*ITS1+2* (61 °C)	F3	TGTTTATTCATCTACCGTCGGGAGG
	B3	CGTTGGGAGCGTGCGTGC
	FIP	CATTGTATGTAAATGTTGTGTCGTGACGGAGGGGATGACCACGGCG
	BIP	GCAAACATGTAACAATGTTGCTGCGGCAAAGGGTCGTCGATGGGTC
	LoopF	CGGGAGTCGCACTTAACAGATTTG
	LoopB	CCGTGATAAAATGGTGGATGAGCC
*ITS2* (65 °C)	F3	GCCTCGTGGTTGGTTGAA
	B3	GCTTAAACTCAGCGGGTAGC
	FIP	GATTGGTCTCGAGCGTGGCTTGGGTTCATGGCCGACTT
	BIP	GTGCGAGCCGGTCAGTTCTGCCTGAGGTCTCGTTGGGAG
	LoopF	TCCACCATTTTATCACGGCG
	LoopB	GACGACCCTTTGCGTGCA
*ORF160b* (62 °C)	F3	CCGAGTCTGCTGCCGTAT
	B3	ATGAGATTGAGTTCCACGCA
	FIP	GGGGTCAGTATTACGCCTCTGACAAAGAAAGAGAGTGACGATG
	biotin-FIP	b-GGGGTCAGTATTACGCCTCTGACAAAGAAAGAGAGTGACGATG
	BIP	TCTGATAGATAGTGGCAAACATTAGTTGCTGCTATTCCATCTATTCAT
	LoopF	TTCTGATTCCGCTCATTGG
	FITC-LoopF	FITC-TTCTGATTCCGCTCATTGG
	LoopB	CAAGATATAGAAGACTATTAGCCCG
*18S rRNA* (94/60/72 °C)	TR03 ^2^ (forward)	TCTGCCCTATCAACTTTCGATGGTA
	TR04 ^2^ (reverse)	AATTTGCGCGCCTGCTGCCTTCCTT

^1^ T_A_: assay reaction temperature; ^2^ Allmann et al., 1993.

**Table 4:** Pre-assessment of sensitivity of the five LAMP assays based on serially diluted soybean DNA (+: 2/2 replicates positive; (+): 1/2 replicates positive; -: 2/2 replicates negative).

Dilution Soy Equivalent (mg/kg)					
	1:10^3^	1:10^5^	1:10^6^	1:10^7^	/
	1000	10	1	0.1	NTC
*atpA*	+	+	(+)	(+)	-
*ITS1*	+	(+)	(+)	(+)	(+)
*ITS1+2*	+	+	+	+	+
*ITS2*	+	+	+	+	-
*ORF160b*	+	+	(+)	-	-

**Table 5:** Results of initial specificity assessment of the LAMP assays *atpA*, *ITS2*, and *ORF160b* with closely related species and species commonly used as food ingredients. The DNA of potential cross-reactants was analyzed undiluted and 100-fold diluted, tofu served as a positive control (+: positive reaction; -: negative reaction).

Sample No.	Common Name (Family)	LAMP Assay					
		*atpA*		*ITS2*		*ORF160b*	
		Undiluted	1:100	Undiluted	1:100	Undiluted	1:100
5	peanut (*Fabaceae*)	-	-	+	+	-	-
6	chickpea (*Fabaceae*)	+	+	-	-	-	-
7	kidney bean (*Fabaceae*)	+	+	-	-	-	-
8	pinto bean (*Fabaceae*)	+	+	-	-	-	-
13	barley (*Poaceae*)	-	-	+	-	-	-
15	wheat (*Poaceae*)	-	-	+	-	-	-
16	brown lentil (*Fabaceae*)	+	+	-	-	-	-
18	pea (*Fabaceae*)	+	+	-	-	-	-
24	red lentil (*Fabaceae*)	+	+	+	+	-	-
26	blue lupine (*Fabaceae*)	-	-	-	+	-	-
27	yellow lupine (*Fabaceae*)	-	-	-	-	-	-
28	white lupine (*Fabaceae*)	-	-	-	-	-	-
34	100% tofu (*Fabaceae*)	+	+	+	+	+	+

**Table 6:** Optimization of FIP:biotin-FIP and LoopF:FITC-LoopF primer ratios for fluorescence-based real-time detection of LAMP using SYBR Green I.

Primer Ratio (FIP:Biotin-FIP; LoopF:FITC-LoopF)	FIP	Biotin-FIP	LoopF	FITC-LoopF
[1] 0:1; 0:1	0 µM	1.6 µM	0 µM	0.4 µM
[2] 0:1; 1:1	0 µM	1.6 µM	0.2 µM	0.2 µM
[3] 1:1; 1:1	0.8 µM	0.8 µM	0.2 µM	0.2 µM
[4] 1:1; 1:3	0.8 µM	0.8 µM	0.3 µM	0.1 µM

**Table 7:** LAMP optimization parameters.

Parameter	Conditions	Optimum	
		*atpA*	*ORF160b*
temperature	58–68 °C	63 °C	62 °C
primer concentration	0.2–1.5×	1× (FIP/BIP 1.6 µM; F3/B3 0.2 µM; LoopF/B 0.4 µM)	0.75× (FIP/BIP 1.2 µM; F3/B3 0.15 µM; LoopF/B 0.3 µM)
polymerase concentration	0.5–1.5×	1× (0.32 U/µl)	1× (0.32 U/µl)
Mg^2+^ concentration	4 mM–10 mM	8 mM	6 mM
enhancer	DMSO, betaine	10% DMSO (1.4 M)	6% DMSO (0.8 M); 0.25 mg/mL BSA

## Supplementary Materials

The following are available online at https://www.mdpi.com/2304-8158/9/4/423/s1. Figure S1: Sequence alignment of the *atpA* gene; Figure S2: Sequence alignment of the genes encoding ribosomal RNA and both internal transcribed spacers, *ITS1* and *ITS2*; Figure S3: Sequence alignment of the mitochondrial genome of soybean (*Glycine max*; nucleotides 205217−205754); sequence of the open reading frame 160b (*ORF160b*; nucleotides 205230−205712).
