# Supplementary material for: The Development of Highly Specific and Sensitive Primers for the Detection of Potentially Allergenic Soybean (Glycine max) Using Loop-Mediated Isothermal Amplification Combined with Lateral Flow Dipstick (LAMP-LFD)
Source: Foods. 2020 Apr 3;9(4):423. doi: 10.3390/foods9040423 (PMC7231045; doi:10.3390/foods9040423)
Supplement: Supplementary file 1 [file foods-09-00423-s001.pdf]

Figure S1. Sequence alignment of the atpA gene. Red: homologous nucleotides in comparison with the reference sequence of soybean (*Glycine max*, GenBank acc. No. Z14031.1); green: newly designed LAMP primers targeting the atpA gene of soybean (*Glycine max*, GenBank acc. No. Z14031.1)

```
                                                     6101                                                                                        F3 atpA              6200
                                                                                                                                                 GATATCTTGGTTTTGGAAAACTG TT
   soybean (Glycine max) atpA Z14031.1     (5791)   ATATGCTTAGTCAAAACCACTCTTGCTACGAAGCTACTTTTTTTTTGAGGGAGTCCGTTTTTCGGGATAGGCAGGATATCTTGGTTTTGGAAAACTGGTT
   soybean (Glycine max) atp1-1 JX463295.1 (5793)   ATATGCTTAGTCAAAACCACTCTTGCTACGAAG-----TTTTTTTTGAGGGAGTCCGTTTTTCGGGATAGGCAGGATATCTTGGTTTTGGAAAACTGGTT
   soybean (Glycine max) atp1-2 JX463295.1 (5790)   ATATGCTTAGTCAAAACCACTCTTGCTACGAAG-----TTTTTTTTGAGGGAGTCCGTTTTTCGGGATAGGCAGGATATCTTGGTTTTGGAAAACTGGTT
   soybean (Glycine max) atp1-3 JX463295.1 (5791)   ATATGCTTAGTCAAAACCACTCTTGCTACGAAG-----TTTTTTTTGAGGGAGTCCGTTTTTCGGGATAGGCAGGATATCTTGGTTTTGGAAAACTGGTT
             pea (Pisum sativum) atpA D14698.1 (4449)   TGAGACTTTCTCGTCGTTAGTTTGCTTGCTTTGTTTCCGGGGAGAGGTGAGCCGCTTACGCTGTGAGGAGACAGGAGCTCTCGAGCTTAGAGAA---GTT
green bean (Phaseolus vulgaris) atpA M64246.1 (2719)   ATTCTGACTTACACTATTCTATTTTCCGGGATTGATGTCCGCTTGGAAAGGAGTAAGGTTTAGGCTTTACCCAGTTGAACTCGAGGTTGGTAATAAACTG

                                                     6201 F2 atpA       LoopF atpA                            F1 atpA                  B1 atpA             6300
                                                     AAAAAGACGAATGCTCCTCCATCTGTGGTTATTGCTTTGGGA             TAACTATTCTAGTTAGCCTGGGCTAAGAAGACCCTGTCAAGCCTT
   soybean (Glycine max) atpA Z14031.1     (5891)   AAAAAGACGAATGCTCCTCCATCTGTGGTTATTGCTTTGGGATCCTATAAGCTACTAACTATTCTAGTTAGCCTGGGCTAAGAAGACCCTGTCAAGCCTT
   soybean (Glycine max) atp1-1 JX463295.1 (5888)   AAAAAGACGAATGCTCCTCCATCTGTGGTTATTGCTTTGGGATCCTATAAGCTACTAACTATTCTAGTTAGCCTGGGCTAAGAAGACCCTGTCAAGCCTT
   soybean (Glycine max) atp1-2 JX463295.1 (5885)   AAAAAGACGAATGCTCCTCCATCTGTGGTTATTGCTTTGGGATCCTATAAGCTACTAACTATTCTAGTTAGCCTGGGCTAAGAAGACCCTGTCAAGCCTT
   soybean (Glycine max) atp1-3 JX463295.1 (5886)   AAAAAGACGAATGCTCCTCCATCTGTGGTTATTGCTTTGGGATCCTATAAGCTACTAACTATTCTAGTTAGCCTGGGCTAAGAAGACCCTGTCAAGCCTT
             pea (Pisum sativum) atpA D14698.1 (4546)   CCCTAGGCCAGACAGAGGTCTAGGGGGAGTCCGGATAGAGGACTCTTTAAACTAAACCGGTGGGCCCGCATATGTAGGAAGGCTTAAGCTT---------
green bean (Phaseolus vulgaris) atpA M64246.1 (2819)   AAGCTGCTCAGTCTTCTAGCATA-GCGGAGTTAGATATATCGAAGAATGAACTTAGCCTTCAACCACAAATGGGGTCCTTTAGGCAGCCGATTGCCCGTT

                                                     6301        LoopB atpA                         B2 atpA                    B3 atpA                 6400
                                                     TG          GGGCAAT-GCCACTTCGAAAG          GGAGAGATCTTTGAACAGCA     GACGGATCTCAGAACAT-AGC
   soybean (Glycine max) atpA Z14031.1     (5991)   TGATTCGACTTTGGGCAAT-GCCACTTCGAAAGAAAAGGAATCGGAGAGATCTTTGAACAGCAGAAAAGACGGATCTCAGAACAT-AGCCATAGTTACGG
   soybean (Glycine max) atp1-1 JX463295.1 (5988)   TGATTCGACTTTGGGCAAT-GCCACTTCGAAAGAAAAGGAATCGGAGAGATCTTTGAACAGCAGAAAAGACGGATCTCAGAACAT-AGCCATAGTTACGG
   soybean (Glycine max) atp1-2 JX463295.1 (5985)   TGATTCGACTTTGGGCAAT-GCCACTTCGAAAGAAAAGGAATCGGAGAGATCTTTGAACAGCAGAAAAGACGGATCTCAGAACAT-AGCCATAGTTACGG
   soybean (Glycine max) atp1-3 JX463295.1 (5986)   TGATTCGACTTTGGGCAAT-GCCACTTCGAAAGAAAAGGAATCGGAGAGATCTTTGAACAGCAGAAAAGACGGATCTCAGAACAT-AGCCATAGTTACGG
             pea (Pisum sativum) atpA D14698.1 (4637)   ----------------------------------------------------------------------------------------------------
green bean (Phaseolus vulgaris) atpA M64246.1 (2918)   TGCTTCCACTGACTTACGTCGTGAACATTTTTATTAATCAATAGAATTCCTCTTCGATCCCATCCTATGAGCCCTGCGAGCTCACGAGTCGTCAGTGATG
```

Figure S2. Sequence alignment of the genes encoding ribosomal RNA and both internal transcribed spacers, ITS1 and ITS2. Red: homologous nucleotides in comparison with the reference sequence of soybean (*Glycine max*, GenBank acc. No. FJ609734.1); yellow: start and end of the 18S, ITS1, 5.8S, ITS2 and the 26S gene, respectively; green: newly designed primers targeting the ITS1 gene of soybean (*Glycine max*); magenta: newly designed primers spanning the ITS1+2 genes of soybean (*Glycine max*); cyan: newly designed LAMP primers targeting the ITS2 gene of soybean (*Glycine max*)

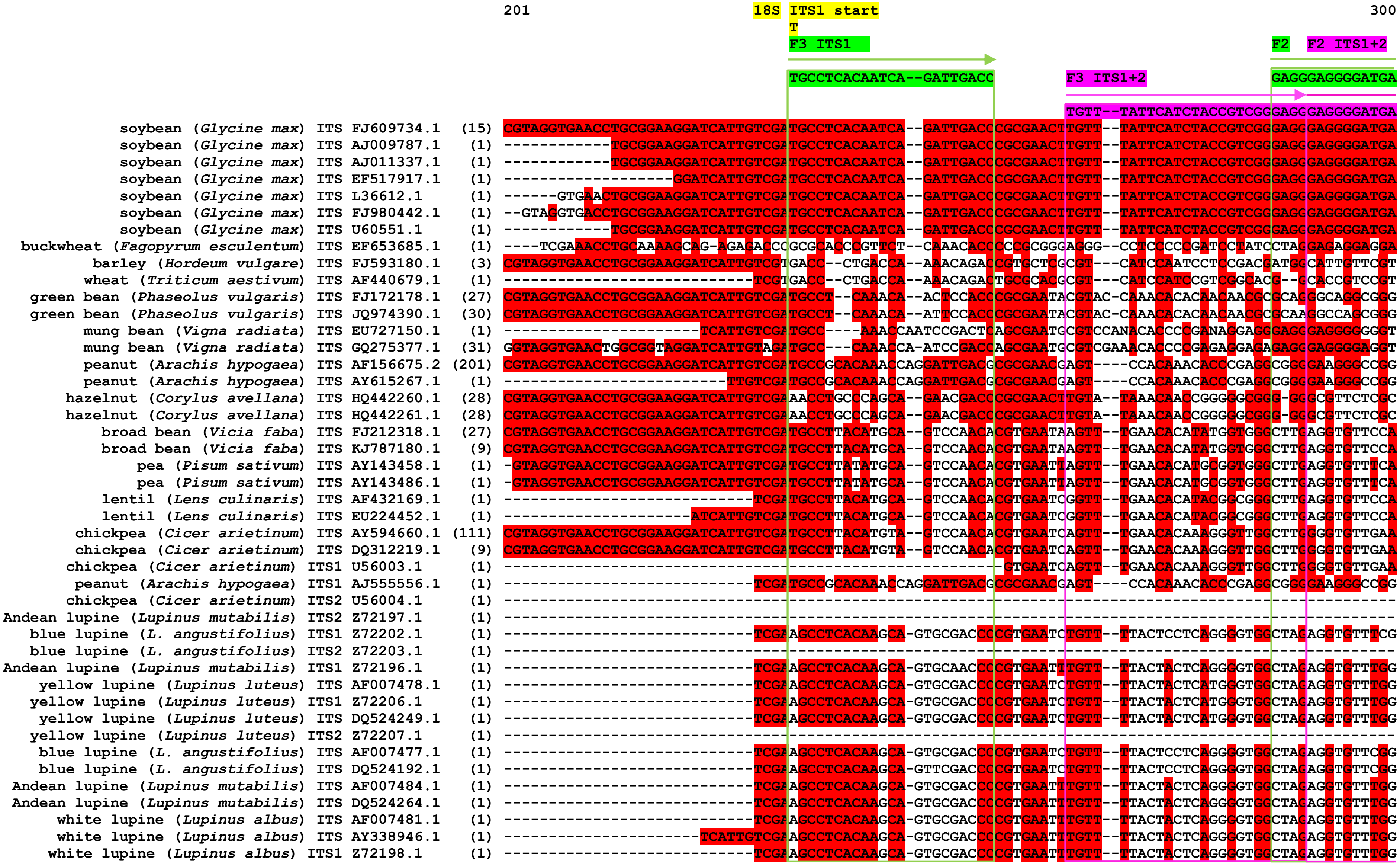

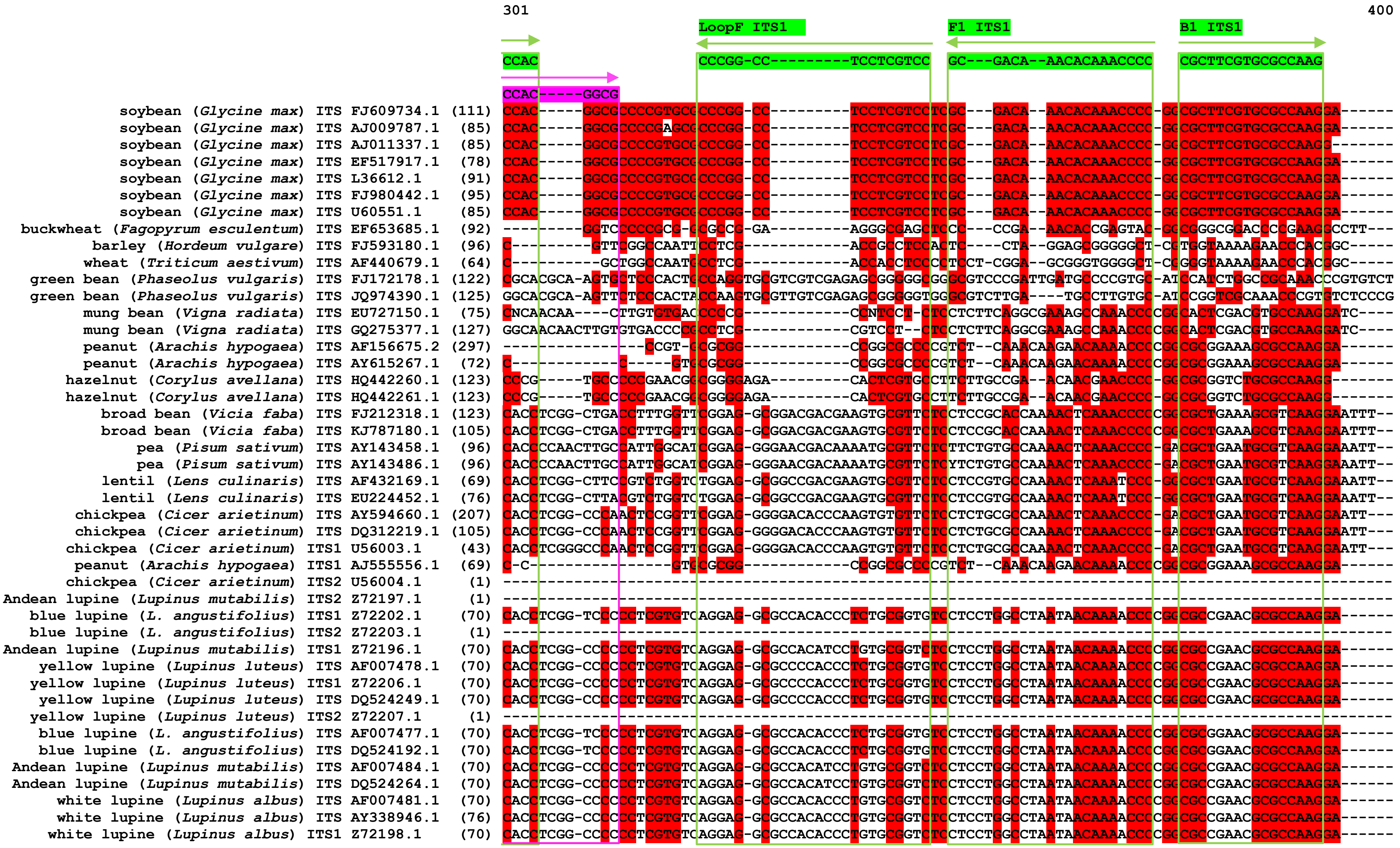
301
400
LoopF ITS1
F1 ITS1
B1 ITS1
CCAC
CCCGG-CC---------TCCTCGTCC
GC---GACA--AACACAAACCCC
CGCTTCGTGCGCCAAG
CCAC-----GGCG
soybean (Glycine max) ITS FJ609734.1 (111)
soybean (Glycine max) ITS AJ009787.1 (85)
soybean (Glycine max) ITS AJ011337.1 (85)
soybean (Glycine max) ITS EF517917.1 (78)
soybean (Glycine max) ITS L36612.1 (91)
soybean (Glycine max) ITS FJ980442.1 (95)
soybean (Glycine max) ITS U60551.1 (85)
buckwheat (Fagopyrum esculentum) ITS EF653685.1 (92)
barley (Hordeum vulgare) ITS FJ593180.1 (96)
wheat (Triticum aestivum) ITS AF440679.1 (64)
green bean (Phaseolus vulgaris) ITS FJ172178.1 (122)
green bean (Phaseolus vulgaris) ITS JQ974390.1 (125)
mung bean (Vigna radiata) ITS EU727150.1 (75)
mung bean (Vigna radiata) ITS GQ275377.1 (127)
peanut (Arachis hypogaea) ITS AF156675.2 (297)
peanut (Arachis hypogaea) ITS AY615267.1 (72)
hazelnut (Corylus avellana) ITS HQ442260.1 (123)
hazelnut (Corylus avellana) ITS HQ442261.1 (123)
broad bean (Vicia faba) ITS FJ212318.1 (123)
broad bean (Vicia faba) ITS KJ787180.1 (105)
pea (Pisum sativum) ITS AY143458.1 (96)
pea (Pisum sativum) ITS AY143486.1 (96)
lentil (Lens culinaris) ITS AF432169.1 (69)
lentil (Lens culinaris) ITS EU224452.1 (76)
chickpea (Cicer arietinum) ITS AY594660.1 (207)
chickpea (Cicer arietinum) ITS DQ312219.1 (105)
chickpea (Cicer arietinum) ITS1 U56003.1 (43)
peanut (Arachis hypogaea) ITS1 AJ555556.1 (69)
chickpea (Cicer arietinum) ITS2 U56004.1 (1)
Andean lupine (Lupinus mutabilis) ITS2 Z72197.1 (1)
blue lupine (L. angustifolius) ITS1 Z72202.1 (70)
blue lupine (L. angustifolius) ITS2 Z72203.1 (1)
Andean lupine (Lupinus mutabilis) ITS1 Z72196.1 (70)
yellow lupine (Lupinus luteus) ITS AF007478.1 (70)
yellow lupine (Lupinus luteus) ITS1 Z72206.1 (70)
yellow lupine (Lupinus luteus) ITS DQ524249.1 (70)
yellow lupine (Lupinus luteus) ITS2 Z72207.1 (1)
blue lupine (L. angustifolius) ITS AF007477.1 (70)
blue lupine (L. angustifolius) ITS DQ524192.1 (70)
Andean lupine (Lupinus mutabilis) ITS AF007484.1 (70)
Andean lupine (Lupinus mutabilis) ITS DQ524264.1 (70)
white lupine (Lupinus albus) ITS AF007481.1 (70)
white lupine (Lupinus albus) ITS AY338946.1 (76)
white lupine (Lupinus albus) ITS1 Z72198.1 (70)

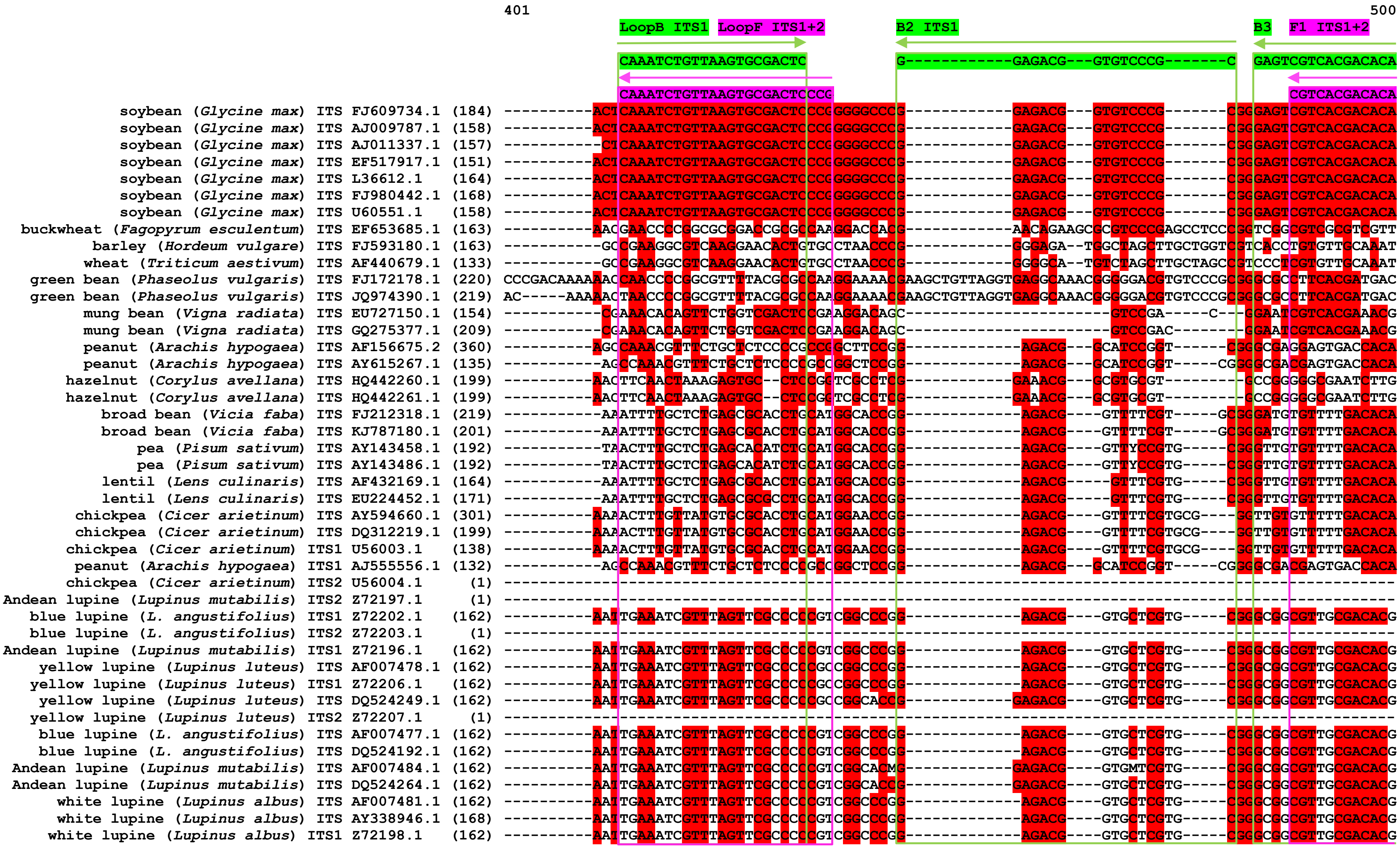

401
500
LoopB ITS1
LoopF ITS1+2
B2 ITS1
B3
F1 ITS1+2
CAAATCTGTTAAGTGCGACTC
G------------GAGACG---GTGTCCCG-------C
GAGTCGTCACGACACA
CAAATCTGTTAAGTGCGACTCCCG
CGTCACGACACA
soybean (Glycine max) ITS FJ609734.1 (184)
soybean (Glycine max) ITS AJ009787.1 (158)
soybean (Glycine max) ITS AJ011337.1 (157)
soybean (Glycine max) ITS EF517917.1 (151)
soybean (Glycine max) ITS L36612.1 (164)
soybean (Glycine max) ITS FJ980442.1 (168)
soybean (Glycine max) ITS U60551.1 (158)
buckwheat (Fagopyrum esculentum) ITS EF653685.1 (163)
barley (Hordeum vulgare) ITS FJ593180.1 (163)
wheat (Triticum aestivum) ITS AF440679.1 (133)
green bean (Phaseolus vulgaris) ITS FJ172178.1 (220)
green bean (Phaseolus vulgaris) ITS JQ974390.1 (219)
mung bean (Vigna radiata) ITS EU727150.1 (154)
mung bean (Vigna radiata) ITS GQ275377.1 (209)
peanut (Arachis hypogaea) ITS AF156675.2 (360)
peanut (Arachis hypogaea) ITS AY615267.1 (135)
hazelnut (Corylus avellana) ITS HQ442260.1 (199)
hazelnut (Corylus avellana) ITS HQ442261.1 (199)
broad bean (Vicia faba) ITS FJ212318.1 (219)
broad bean (Vicia faba) ITS KJ787180.1 (201)
pea (Pisum sativum) ITS AY143458.1 (192)
pea (Pisum sativum) ITS AY143486.1 (192)
lentil (Lens culinaris) ITS AF432169.1 (164)
lentil (Lens culinaris) ITS EU224452.1 (171)
chickpea (Cicer arietinum) ITS AY594660.1 (301)
chickpea (Cicer arietinum) ITS DQ312219.1 (199)
chickpea (Cicer arietinum) ITS1 U56003.1 (138)
peanut (Arachis hypogaea) ITS1 AJ555556.1 (132)
chickpea (Cicer arietinum) ITS2 U56004.1 (1)
Andean lupine (Lupinus mutabilis) ITS2 Z72197.1 (1)
blue lupine (L. angustifolius) ITS1 Z72202.1 (162)
blue lupine (L. angustifolius) ITS2 Z72203.1 (1)
Andean lupine (Lupinus mutabilis) ITS1 Z72196.1 (162)
yellow lupine (Lupinus luteus) ITS AF007478.1 (162)
yellow lupine (Lupinus luteus) ITS1 Z72206.1 (162)
yellow lupine (Lupinus luteus) ITS DQ524249.1 (162)
yellow lupine (Lupinus luteus) ITS2 Z72207.1 (1)
blue lupine (L. angustifolius) ITS AF007477.1 (162)
blue lupine (L. angustifolius) ITS DQ524192.1 (162)
Andean lupine (Lupinus mutabilis) ITS AF007484.1 (162)
Andean lupine (Lupinus mutabilis) ITS DQ524264.1 (162)
white lupine (Lupinus albus) ITS AF007481.1 (162)
white lupine (Lupinus albus) ITS AY338946.1 (168)
white lupine (Lupinus albus) ITS1 Z72198.1 (162)

```
                                                            501     5.8S start                                                                      600
                                                                    A
                                                            ACATTT
                                                            ACATTTACATACAATG
soybean (Glycine max) ITS FJ609734.1 (252)                  ACATTTACATACAATGACTCTCGGCAACGGATATCTCGGCTCTTGCATCGATGAAGAACGTAGCGAAATGCGATACTTGGTGTGAATTGCA-GAATCCCG
soybean (Glycine max) ITS AJ009787.1 (226)                  ACATTTACATACAATGACTCTCGGCAACGGATATCTCGGCTCTTGCATCGATGAAGAACGTAGCAAAATGCGATACTTGGTGTAAATTGCA-GAATCCCG
soybean (Glycine max) ITS AJ011337.1 (224)                  ACATTTACATACAATGACTCTCGGCAACGGATATCTCGGCTCTTGCATCGATGAAGAACGTAGCGAAATGCGATACTTGGTGTAAATTGCA-GAATCCCG
soybean (Glycine max) ITS EF517917.1 (219)                  ACATTTACATACAATGACTCTCGGCAACGGATATCTCGGCTCTTGCATCGATGAAGAACGTAGCGAAATGCGATACTTGGTGTGAATTGCA-GAATCCCG
soybean (Glycine max) ITS L36612.1 (232)                    ACATTTACATACAATGACTCTCGGCAACGGATATCTCGGCTCTTGCATCGATGAAGAACGTAGCGAAATGCGATACTTGGTGTGAATTGCA-GAATCCCG
soybean (Glycine max) ITS FJ980442.1 (236)                  ACATTTACATACAATGACTCTCGGCAACGGATATCTCGGCTCTTGCATCGATGAAGAACGTAGCGAAATGCGATACTTGGTGTGAATTGCA-GAATCCCG
soybean (Glycine max) ITS U60551.1 (226)                    ACATTTACATACAATGACTCTCGGCAACGGATATCTCGGCTCTTGCATCGATGAAGAACGTAGCGAAATGCGATACTTGGTGTGAATTGCA-GAATCCCG
buckwheat (Fagopyrum esculentum) ITS EF653685.1 (241)       TCTACGAAACAGAACGACTCTCGGCAACGGATATCTCGGCTCTCGCATCGATGAAGAACGTAGCGAAATGCGATACTTGGTGTGAATTGCA-GAATCCCG
barley (Hordeum vulgare) ITS FJ593180.1 (238)               ATATTTAATCCACACGACTCTCGGCAACGGATATCTCGGCTCTCGCATCGATGAAGAACGTAGCGAAATGCGATACCTGGTGTGAATTGCA-GAATCCCG
wheat (Triticum aestivum) ITS AF440679.1 (208)              CTATTTAATCCACACGACTCTCGGCAACGGATATCTCGGCTCTCGCATCGATGAAGAACGTAGCGAAATGCGATACCTGGTGTGAATTGCA-GAATCCCG
green bean (Phaseolus vulgaris) ITS FJ172178.1 (320)        ATGTT-ATGTAAAATGACTCTCGGCAACGGATATCTCGGCTCTTGCATCGATGAAGAACGTAGCGAAATGCGATACTTGGTGTGAATTGCA-GAATCCCG
green bean (Phaseolus vulgaris) ITS JQ974390.1 (314)        ATGTT-ATGTAAAATGACTCTCGGCAACGGATATCTCGGCTCTTGCATCGATGAAGAACGTAGCGAAATGCGATACTTGGTGTGAATTGCA-GAATCCCG
mung bean (Vigna radiata) ITS EU727150.1 (212)             AATCA------AAACGACTCTCGGCAACGGATATCTCGGCTCTTGCATCGATGAAGAACGTAGCGAAATGCGATACTTGGTGTGAATTGCA-GAATCCCG
mung bean (Vigna radiata) ITS GQ275377.1 (267)             AATCA------AAACGACTCTCGGCAACGGATATCTCGGCTCTTGCATCGATGAAGAACGTAGCGAAATGCGATACTTGGTGTGAATTGCA-GAATCCCG
peanut (Arachis hypogaea) ITS AF156675.2 (428)             AGAGTTAA--AGAACGACTCTCGGCAACGGATATCTCG-CTCTTGCATCGATGAAGAACGTAGCGAAATGCGATACTTGGTGTGAATTGCAAGAATCCCG
peanut (Arachis hypogaea) ITS AY615267.1 (203)             AGAGTTA---AGAACGACTCTCGGCAACGGATATCTCGGCTCTTGCATCGATGAAGAACGTAGCGAAATGCGATACTTGGTGTGAATTGCA-GAATCCCG
hazelnut (Corylus avellana) ITS HQ442260.1 (263)           TGCAA-AACCATAACGACTCTCGGCAACGGATATCTCGGCTCTCGCATCGATGAAGAACGTAGCGAAATGCGATACTTGGTGTGAATTGCA-GAATCCCG
hazelnut (Corylus avellana) ITS HQ442261.1 (263)           TGCAA-AACCATAACGACTCTCGGCAACGGATATCTCGGCTCTCGCATCGATGAAGAACGTAGCGAAATGCGATACTTGGTGTGAATTGCA-GAATCCCG
broad bean (Vicia faba) ITS FJ212318.1 (286)                TGATA----TAGAATGACTCTCGGCAACGGATATCTAGGCTCTTGCATCGATGAAGAACGTAGCGAAATGCGATACTTGGTGTGAATTGCA-GAATCCCG
broad bean (Vicia faba) ITS KJ787180.1 (268)                TGATA----TAGAATGACTCTCGGCAACGGATATCTAGGCTCTTGCATCGATGAAGAACGTAGCGAAATGCGATACTTGGTGTGAATTGCA-GAATCCCG
pea (Pisum sativum) ITS AY143458.1 (259)                    TTAAT---ATAAAATGACTCTCGGCAACGGATATCTAGGCTCTTGCATCGATGAAGAACGTAGCGAAATGCGATACTTGGTGTGAATTGCA-GAATCCCG
pea (Pisum sativum) ITS AY143486.1 (259)                    TTAAT---ATAAAATGACTCTCGGCAACGGATATCTAGGCTCTTGCATCGATGAAGAACGTAGCGAAATGCGATACTTGGTGTGAATTGCA-GAATCCCG
lentil (Lens culinaris) ITS AF432169.1 (230)                TGATA----TAGAATGACTCTCGGCAACGGATATCTAGGCTCTTGCATCGATGAAGAACGTAGCGAAATGCGATACTTGGTGTGAATTGCA-GAATCCCG
lentil (Lens culinaris) ITS EU224452.1 (237)                TGATA----TAGAATGACTCTCGGCAACGGATATCTAGGCTCTTGCATCGATGAAGAACGTAGCGAAATGCGATACTTGGTGTGAATTGCA-GAATCCCG
chickpea (Cicer arietinum) ITS AY594660.1 (370)             TGAAA-ATATAGAATGACTCTCGGCAACGGATATCTAGGCTCTTGCATCGATGAAGAACGTAGCGAAATGCGATACTTGGTGTGAATTGCA-GAATCCCG
chickpea (Cicer arietinum) ITS DQ312219.1 (268)             TGAAATA--TAGAATGACTCTCGGCAACGGATATCTAGGCTCTTGCATCGATGAAGAACGTAGCGAAATGCGATACTTGGTGTGAATTGCA-GAATCCCG
chickpea (Cicer arietinum) ITS1 U56003.1 (207)              TGAAATAT--------------------------------------------------------------------------------------------
peanut (Arachis hypogaea) ITS1 AJ555556.1 (200)             AGA-------------------------------------------------------------------------------------------------
chickpea (Cicer arietinum) ITS2 U56004.1 (1)                ----------------------------------------------------------------------------------------------------
Andean lupine (Lupinus mutabilis) ITS2 Z72197.1 (1)         ----------------------------------------------------------------------------------------------------
blue lupine (L. angustifolius) ITS1 Z72202.1 (230)          CTTAT----CCTAA--------------------------------------------------------------------------------------
blue lupine (L. angustifolius) ITS2 Z72203.1 (1)            ----------------------------------------------------------------------------------------------------
Andean lupine (Lupinus mutabilis) ITS1 Z72196.1 (230)       CTTAT----CCTAA--------------------------------------------------------------------------------------
yellow lupine (Lupinus luteus) ITS AF007478.1 (230)         CTTAT----CCTAAAGACTCTCGGCAACGGATATCTCGGCTCTTGCATCGATGAAGAACGTAGCGAAATGCGATACTTGGTGTGAATTGCA-GAATCCCG
yellow lupine (Lupinus luteus) ITS1 Z72206.1 (230)          CTTAT----CCTAA--------------------------------------------------------------------------------------
yellow lupine (Lupinus luteus) ITS DQ524249.1 (231)         CTTAT----CCTAAAGACTCTCGGCAACGGATATCTCGGCTCTTGCATCGATGAAGAACGTAGCGAAATGCGATACTTGGTGTGAATTGCA-GAATCCCG
yellow lupine (Lupinus luteus) ITS2 Z72207.1 (1)            ----------------------------------------------------------------------------------------------------
blue lupine (L. angustifolius) ITS AF007477.1 (230)         CTTAT----CCTAAAGACTCTCGGCAACGGATATCTCGGCTCTTGCATCGATGAAGAACGTAGCGAAATGCGATACTTGGTGTGAATTGCA-GAATCCCG
blue lupine (L. angustifolius) ITS DQ524192.1 (230)         CTTAT----CCTAAAGACTCTCGGCAACGGATATCTCGGCTCTTGCATCGATGAAGAACGTAGCGAAATGCGATACTTGGTGTGAATTGCA-GAATCCCG
Andean lupine (Lupinus mutabilis) ITS AF007484.1 (231)       CTTAT----CCTAAAGACTCTCGGCAACGGATATCTCGGCTCTTGCATCGATGAAGAACGTAGCGAAATGCGATACTTGGTGTGAATTGCA-GAATCCCG
Andean lupine (Lupinus mutabilis) ITS DQ524264.1 (231)       CTTAT----CCTAAAGACTCTCGGCAACGGATATCTCGGCTCTTGCATCGATGAAGAACGTAGCGAAATGCGATACTTGGTGTGAATTGCA-GAATCCCG
white lupine (Lupinus albus) ITS AF007481.1 (230)           CTTAT----CCTAAAGACTCTCGGCAACGGATATCTCGGCTCTTGCATCGATGAAGAACGTAGCGAAATGCGATACTTGGTGTGAATTGCA-GAATCCCG
white lupine (Lupinus albus) ITS AY338946.1 (236)           CTTAT----CCTAAAGACTCTCGGCAACGGATATCTCGGCTCTTGCATCGATGAAGAACGTAGCGAAATGCGATACTTGGTGTGAATTGCA-GAATCCCG
white lupine (Lupinus albus) ITS1 Z72198.1 (230)            CTTAT----CCTAA--------------------------------------------------------------------------------------
```

Species	Region	Accession	Start	601 … 700 (ITS2 start: C; B1 ITS1+2: GCAAACATG)
soybean (*Glycine max*)	ITS	FJ609734.1	(351)	TGAACCATCGAGTCTTTGAACGCAAGTTGCGCCCGAAGCCATT-AGGCCGAGGGCACGCCTGCCTGGGTGTCACACATCGTTTCCCCAA-CGCAAACATG
soybean (*Glycine max*)	ITS	AJ009787.1	(325)	TAAACCATCGAGTCTTTAAACGCAAGTTGCGCCCGAAGCCATT-AGGCCGAGGGCACGCCTGCCTGGGTGTCACACATCGTTTCCCCAA-CGCAAACATG
soybean (*Glycine max*)	ITS	AJ011337.1	(323)	TAAACCATCGAG-CTTTGAACGCAAGTTGCGCCCGAAGCCATTTAGGCCGAGGGCACGCCTGCCTGGGTGTCACACATCGTTTCCCCAA-CGCAAACATG
soybean (*Glycine max*)	ITS	EF517917.1	(318)	TGAACCATCGAGTCTTTGAACGCAAGTTGCGCCCGAAGCCATT-AGGCCGAGGGCACGCCTGCCTGGGTGTCACACATCGTTTCCCCAA-CGCAAACATG
soybean (*Glycine max*)	ITS	L36612.1	(331)	TGAACCATCGAGTCTTTGAACGCAAGTTGCGCCCGAAGCCATT-AGGCCGAGGGCACGCCTGCCTGGGTGTCACACATCGTTTCCCCAA-CGCAAACATG
soybean (*Glycine max*)	ITS	FJ980442.1	(335)	TGAACCATCGAGTCTTTGAACGCAAGTTGCGCCCGAAGCCATT-AGGCCGAGGGCACGCCTGCCTGGGTGTCACACATCGTTTCCCCAA-CGCAAACATG
soybean (*Glycine max*)	ITS	U60551.1	(325)	TGAACCATCGAGTCTTTGAACGCAAGTTGCGCCCGAAGCCATT-AGGCCGAGGGCACGCCTGCCTGGGTGTCACACATCGTTTCCCCAA-CGCAAACATG
buckwheat (*Fagopyrum esculentum*)	ITS	EF653685.1	(340)	TGAACCATCGAGTCTTTGAACGCAAGTTGCGCCCGAGGCCTTC-CGGCTGAGGGCACGCCTGTCTGGGCGTCACGCACCGCGTCGCCCC-CTCCCCCTCC
barley (*Hordeum vulgare*)	ITS	FJ593180.1	(337)	CGAACCATCGAGTCTTTGAACGCAAGTTGCGCCCGAGGCCACT-CGGCCGAGGGCACGCCTGCCTGGGCGTCACGCCAAAACACGCTCCCAACCACCCTC
wheat (*Triticum aestivum*)	ITS	AF440679.1	(307)	CGAACCATCGAGTCTTTGAACGCAAGTTGCGCCCGAGGCCACT-CGGCCGAGGGCACGCCTGCCTGGGCGTCACGCCAAAACACGCTCCCAGCCACTCTC
green bean (*Phaseolus vulgaris*)	ITS	FJ172178.1	(418)	TGAACCATCGAGTCTTTGAACGCAAGTTGCGCCCAAAGCCATT-AGGCTGAGGGCACGCCTGCCTGGGTGTCACACATCGTCACCCCCTTCCTTCACACT
green bean (*Phaseolus vulgaris*)	ITS	JQ974390.1	(412)	TGAACCATCGAGTCTTTGAACGCAAGTTGCGCCCAAAGCCATT-AGGCTGAGGGCACGCCTGCCTGGGTGTCACACATCGTCACCCCTTCCTCCACACTT
mung bean (*Vigna radiata*)	ITS	EU727150.1	(305)	TGAACCATCGAGTCTTTGAACGCAAGTTGCGCCCAAAGCCATT-AGGCCGAGGGCACGCCTGCCTGGGTGTCACACATCGTCATCCCCATGCAAAAGCGC
mung bean (*Vigna radiata*)	ITS	GQ275377.1	(360)	TGAACCATCGAGTCTTTGAACGCAAGTTGCGCCCAAAGCCATT-AGGCCGAGGGCACGCCTGCCTGGGTGTCACACATCGTCATCCCCATGCAAAAGCGC
peanut (*Arachis hypogaea*)	ITS	AF156675.2	(525)	TGAACCATCGAGTCTTTGAACGCAAGTTGCGCCCGAAGCCCTTAGGCTGGAGGGCACGCCTGCCTGGGTGTCAACCAAAAGGCGCCCCCCCGTCTCGCCC
peanut (*Arachis hypogaea*)	ITS	AY615267.1	(299)	TGAACCATCGAGTCTTTGAACGCAAGTTGCGCCCGAAGCCCTT-AGGCTGAGGGCACGCCTGCCTGGGTGTCACCAAAAGGCGCCCCCCCGTCTCGCCCG
hazelnut (*Corylus avellana*)	ITS	HQ442260.1	(361)	CGAATCATCGAGTCTTTGAACGCAAGTTGCGCCCGAAGCCATC-TGGTCGAGGGCACGTCTGCCTGGGTGTCACGCATCGTTGCCCCCAACCCCATCGCC
hazelnut (*Corylus avellana*)	ITS	HQ442261.1	(361)	CGAATCATCGAGTCTTTGAACGCAAGTTGCGCCCGAAGCCATC-TGGTCGAGGGCACGTCTGCCTGGGTGTCACGCATCGTTGCCCCCAACCCCATCGCC
broad bean (*Vicia faba*)	ITS	FJ212318.1	(381)	TGAACCATCGAGTCTTTGAACGCAAGTTGCGCCCGATGCCATT-AGGTTGAGGGCACGTCTGCCTGGGTGTCACATATCGAAGCCTCCT-TGCCAATTTC
broad bean (*Vicia faba*)	ITS	KJ787180.1	(363)	TGAACCATCGAGTCTTTGAACGCAAGTTGCGCCCGATGCCATT-AGGTTGAGGGCACGTCTGCCTGGGTGTCACATATCGAAGCCTCCT-TGCCAATTTC
pea (*Pisum sativum*)	ITS	AY143458.1	(355)	TGAACCATCGAGTCTTTGAACGCAAGTTGCGCCCGATGCCATT-AGGTTGAGGGCACGTCTGCCTGGGTGTCACATATTGAAGCCTCCT-GCCAATTTCC
pea (*Pisum sativum*)	ITS	AY143486.1	(355)	TGAACCATCGAGTCTTTGAACGCAAGTTGCGCCCGATGCCATT-AGGTTGAGGGCACGTCTGCCTGGGTGTCACATATTGAAGCCTCCT-GCCAATTTCC
lentil (*Lens culinaris*)	ITS	AF432169.1	(325)	TGAACCATCGAGTCTTTGAACGCAAGTTGCGCCCGATGCCATT-AGGTTGAGGGCACGTCTGCCTGGGTGTCACATATCGAAGCCTCCT-GCCAATTTCC
lentil (*Lens culinaris*)	ITS	EU224452.1	(332)	TGAACCATCGAGTCTTTGAACGCAAGTTGCGCCCGATGCCATT-AGGTTGAGGGCACGTCTGCCTGGGTGTCACATATCGAAGCCTCCT-GCCAATTTCC
chickpea (*Cicer arietinum*)	ITS	AY594660.1	(468)	TGAACCATCGAGTCTTTGAACGCAAGTTGCGCCCGATGCCATT-AGGTTGAGGGCACGTCTGCCTGGGTGTCACATATCGAAGCCCATT-GCCGATTTCC
chickpea (*Cicer arietinum*)	ITS	DQ312219.1	(365)	TGAACCATCGAGTCTTTGAACGCAAGTTGCGCCCGATGCCATT-AGGTTGAGGGCACGTCTGCCTGGGTGTCACATATCGAAGCCCATT-GCCGATTTCC
chickpea (*Cicer arietinum*)	ITS1	U56003.1	(215)	--
peanut (*Arachis hypogaea*)	ITS1	AJ555556.1	(203)	--
chickpea (*Cicer arietinum*)	ITS2	U56004.1	(1)	--ATCGAAGCCCATT-GCCGATTTCC
Andean lupine (*Lupinus mutabilis*)	ITS2	Z72197.1	(1)	---CATCGTTGCCCCCGTGCCTTGGCCA
blue lupine (*L. angustifolius*)	ITS1	Z72202.1	(240)	--
blue lupine (*L. angustifolius*)	ITS2	Z72203.1	(1)	---CATCGTTGCCCCCGTGCCTTGGCCA
Andean lupine (*Lupinus mutabilis*)	ITS1	Z72196.1	(240)	--
yellow lupine (*Lupinus luteus*)	ITS	AF007478.1	(325)	TGAACCATCGAGTCTTTGAACGCAAGTTGCGCCCGAAGCCATT-AGGCCGAGGGCACGCCTGCCTGGGTGTTGCACATCGTTGCCCCCGTGCCTTGGCCA
yellow lupine (*Lupinus luteus*)	ITS1	Z72206.1	(240)	--
yellow lupine (*Lupinus luteus*)	ITS	DQ524249.1	(326)	TGAACCATCGAGTCTTTGAACGCAAGTTGCGCCCGAAGCCATT-AGGCCGAGGGCACGCCTGCCTGGGTGTTGCACATCGTTGCCCCCGTGCCTTGGCCA
yellow lupine (*Lupinus luteus*)	ITS2	Z72207.1	(1)	---CATCGTTGCCCCCGTGCCTTGGCCA
blue lupine (*L. angustifolius*)	ITS	AF007477.1	(325)	TGAACCATCGAGTCTTTGAACGCAAGTTGCGCCCGAAGCCATT-AGGCCGAGGGCACGCCTGCCTGGGTGTTGCACATCGTTGCCCCGT-GCCTTGGCCA
blue lupine (*L. angustifolius*)	ITS	DQ524192.1	(325)	TGAACCATCGAGTCTTTGAACGCAAGTTGCGCCCGAAGCCATT-AGGCCGAGGGCACGCCTGCCTGGGTGTTGCACATCGTTGCCCCGT-GCCTTGGCCA
Andean lupine (*Lupinus mutabilis*)	ITS	AF007484.1	(326)	TGAACCATCGAGTCTTTGAACGCAAGTTGCGCCCGAAGCCATT-AGGCCGAGGGCACGCCTGCCTGGGTGTTGCACATCGTTGCCCCCGTGCCTTGGCCA
Andean lupine (*Lupinus mutabilis*)	ITS	DQ524264.1	(326)	TGAACCATCGAGTCTTTGAACGCAAGTTGCGCCCGAAGCCATT-AGGCCGAGGGCACGCCTGCCTGGGTGTTGCACATCGTTGCCCCCGTGCCTTGGCCA
white lupine (*Lupinus albus*)	ITS	AF007481.1	(325)	TGAACCATCGAGTCTTTGAACGCAAGTTGCGCCCGAAGCCATT-AGGCCGAGGGCACGCCTGCCTGGGTGTTGCACATCGTTGCCCCCGTGCCTTGGCCA
white lupine (*Lupinus albus*)	ITS	AY338946.1	(331)	TGAACCATCGAGTCTTTGAACGCAAGTTGCGCCCGAAGCCATT-AGGCCGAGGGCACGCCTGCCTGGGTGTTGCACATCGTTGCCCCCGTGCCTTGGCCA
white lupine (*Lupinus albus*)	ITS1	Z72198.1	(240)	--

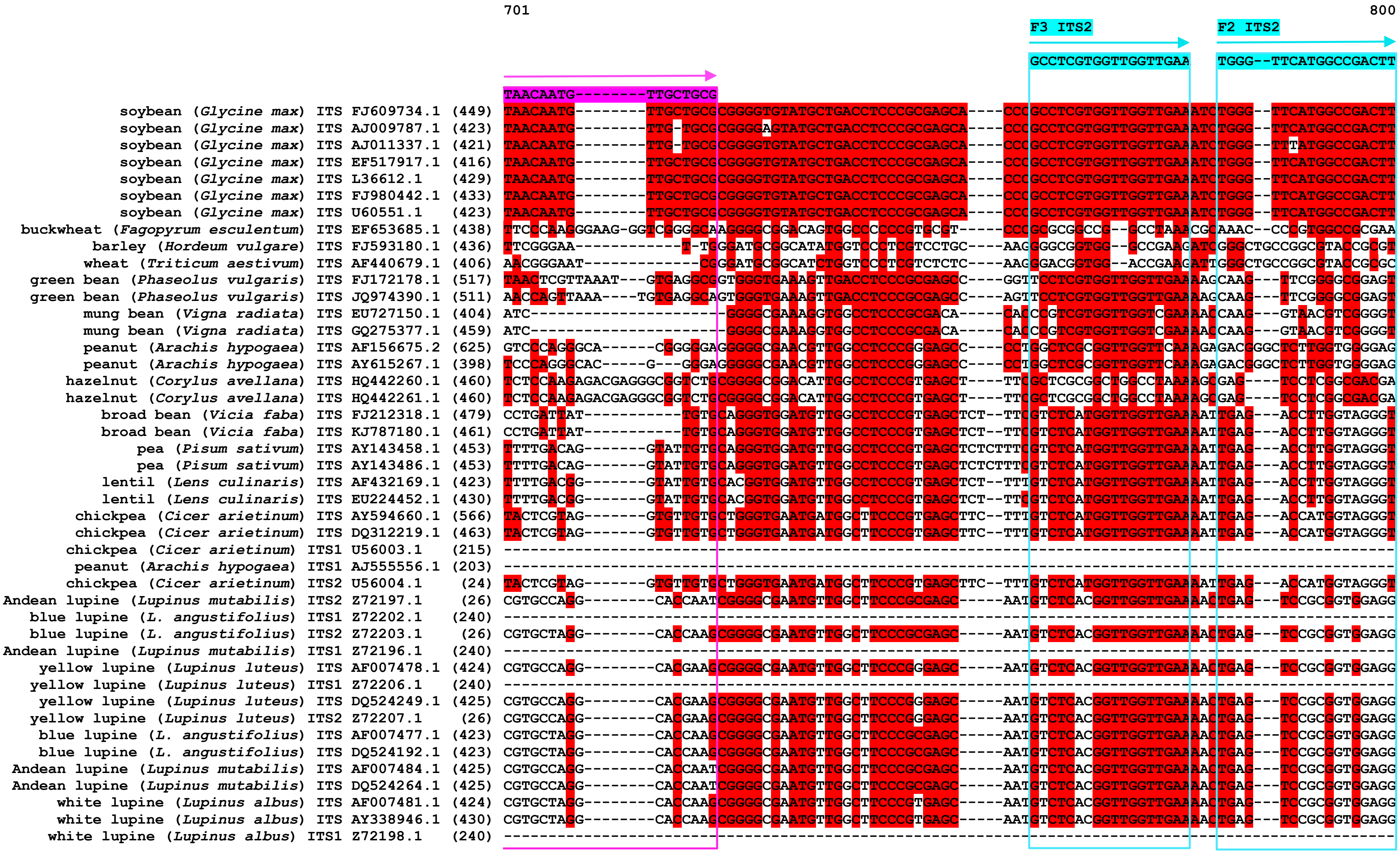

701
800
F3 ITS2
F2 ITS2
GCCTCGTGGTTGGTTGAA
TGGG--TTCATGGCCGACTT
TAACAATG--------TTGCTGCG
soybean (Glycine max) ITS FJ609734.1 (449) TAACAATG--------TTGCTGCGCGGGGTGTATGCTGACCTCCCGCGAGCA----CCCGCCTCGTGGTTGGTTGAAATCTGGG--TTCATGGCCGACTT
soybean (Glycine max) ITS AJ009787.1 (423) TAACAATG--------TTG-TGCGCGGGGAGTATGCTGACCTCCCGCGAGCA----CCCGCCTCGTGGTTGGTTGAAATCTGGG--TTCATGGCCGACTT
soybean (Glycine max) ITS AJ011337.1 (421) TAACAATG--------TTG-TGCGCGGGGTGTATGCTGACCTCCCGCGAGCA----CCCGCCTCGTGGTTGGTTGAAATCTGGG--TTTATGGCCGACTT
soybean (Glycine max) ITS EF517917.1 (416) TAACAATG--------TTGCTGCGCGGGGTGTATGCTGACCTCCCGCGAGCA----CCCGCCTCGTGGTTGGTTGAAATCTGGG--TTCATGGCCGACTT
soybean (Glycine max) ITS L36612.1 (429) TAACAATG--------TTGCTGCGCGGGGTGTATGCTGACCTCCCGCGAGCA----CCCGCCTCGTGGTTGGTTGAAATCTGGG--TTCATGGCCGACTT
soybean (Glycine max) ITS FJ980442.1 (433) TAACAATG--------TTGCTGCGCGGGGTGTATGCTGACCTCCCGCGAGCA----CCCGCCTCGTGGTTGGTTGAAATCTGGG--TTCATGGCCGACTT
soybean (Glycine max) ITS U60551.1 (423) TAACAATG--------TTGCTGCGCGGGGTGTATGCTGACCTCCCGCGAGCA----CCCGCCTCGTGGTTGGTTGAAATCTGGG--TTCATGGCCGACTT
buckwheat (Fagopyrum esculentum) ITS EF653685.1 (438) TTCCCAAGGGAAG-GGTCGGGGCAAGGGGCGGACAGTGGCCCCCCGTGCGT-----CCCGCGCGGCCG--GCCTAAACGCAAAC--CCCGTGGCCGCGAA
barley (Hordeum vulgare) ITS FJ593180.1 (436) TTCGGGAA-----------T-TGGGATGCGGCATATGGTCCCTCGTCCTGC----AAGGGGCGGTGG--GCCGAAGATCGGCTGCCGGCGTACCGCGT
wheat (Triticum aestivum) ITS AF440679.1 (406) AACGGGAAT------------CGGGATGCGGCATCTGGTCCCTCGTCTCTC----AAGGGACGGTGG--ACCGAAGATTGGCTGCCGGCGTACCGCGC
green bean (Phaseolus vulgaris) ITS FJ172178.1 (517) TAACTCGTTAAAT---GTGAGGCGGTGGGTGAAAGTTGACCTCCCGCGAGCC----GGTTCCTCGTGGTTGGTTGAAAAGCAAG---TTCGGGGCGGAGT
green bean (Phaseolus vulgaris) ITS JQ974390.1 (511) AACCAGTTAAA----TGTGAGGCAGTGGGTGAAAGTTGACCTCCCGCGAGCC----AGTTCCTCGTGGTTGGTTGAAAAGCAAG---TTCGGGGCGGAGT
mung bean (Vigna radiata) ITS EU727150.1 (404) ATC---------------------GGGGCGAAAGGTGGCCTCCCGCGACA-----CACCCGTCGTGGTTGGTCGAAAACCAAG---GTAACGTCGGGGT
mung bean (Vigna radiata) ITS GQ275377.1 (459) ATC---------------------GGGGCGAAAGGTGGCCTCCCGCGACA-----CACCCGTCGTGGTTGGTCGAAAACCAAG---GTAACGTCGGGGT
peanut (Arachis hypogaea) ITS AF156675.2 (625) GTCCCAGGGCA------CGGGGGAGGGGGCGAACGTTGGCCTCCCGGGAGCC----CCTGGCTCGCGGTTGGTTCAAAGAGACGGGCTCTTGGTGGGAG
peanut (Arachis hypogaea) ITS AY615267.1 (398) TCCCAGGGCAC-----G---GGGAGGGGGCGAACGTTGGCCTCCCGGGAGCC----CCTGGCTCGCGGTTGGTTCAAAGAGACGGGCTCTTGGTGGGAG
hazelnut (Corylus avellana) ITS HQ442260.1 (460) TCTCCAAGAGACGAGGGCGGTCTGCGGGGCGGACATTGGCCTCCCGTGAGCT----TTCGCTCGCGGCTGGCCTAAAAGCGAG----TCCTCGGCGACGA
hazelnut (Corylus avellana) ITS HQ442261.1 (460) TCTCCAAGAGACGAGGGCGGTCTGCGGGGCGGACATTGGCCTCCCGTGAGCT----TTCGCTCGCGGCTGGCCTAAAAGCGAG----TCCTCGGCGACGA
broad bean (Vicia faba) ITS FJ212318.1 (479) CCTGATTAT-----------TGTGCAGGGTGGATGTTGGCCTCCCGTGAGCTCT--TTCGTCTCATGGTTGGTTGAAAAATTGAG---ACCTTGGTAGGGT
broad bean (Vicia faba) ITS KJ787180.1 (461) CCTGATTAT-----------TGTGCAGGGTGGATGTTGGCCTCCCGTGAGCTCT--TTCGTCTCATGGTTGGTTGAAAAATTGAG---ACCTTGGTAGGGT
pea (Pisum sativum) ITS AY143458.1 (453) TTTTGACAG-------GTATTGTGCAGGGTGGATGTTGGCCTCCCGTGAGCTCTCTTTCGTCTCATGGTTGGTTGAAAAATTGAG---ACCTTGGTAGGGT
pea (Pisum sativum) ITS AY143486.1 (453) TTTTGACAG-------GTATTGTGCAGGGTGGATGTTGGCCTCCCGTGAGCTCTCTTTCGTCTCATGGTTGGTTGAAAAATTGAG---ACCTTGGTAGGGT
lentil (Lens culinaris) ITS AF432169.1 (423) TTTTGACGG-------GTATTGTGCACGGTGGATGTTGGCCTCCCGTGAGCTCT--TTTGTCTCATGGTTGGTTGAAAAATTGAG---ACCTTGGTAGGGT
lentil (Lens culinaris) ITS EU224452.1 (430) TTTTGACGG-------GTATTGTGCACGGTGGATGTTGGCCTCCCGTGAGCTCT--TTCGTCTCATGGTTGGTTGAAAAATTGAG---ACCTTGGTAGGGT
chickpea (Cicer arietinum) ITS AY594660.1 (566) TACTCGTAG-------GTGTTGTGCTGGGTGAATGATGGCTTCCCGTGAGCTTC--TTTGTCTCATGGTTGGTTGAAAAATTGAG---ACCATGGTAGGGT
chickpea (Cicer arietinum) ITS DQ312219.1 (463) TACTCGTAG-------GTGTTGTGCTGGGTGAATGATGGCTTCCCGTGAGCTTC--TTTGTCTCATGGTTGGTTGAAAAATTGAG---ACCATGGTAGGGT
chickpea (Cicer arietinum) ITS1 U56003.1 (215) --
peanut (Arachis hypogaea) ITS1 AJ555556.1 (203) --
chickpea (Cicer arietinum) ITS2 U56004.1 (24) TACTCGTAG-------GTGTTGTGCTGGGTGAATGATGGCTTCCCGTGAGCTTC--TTTGTCTCATGGTTGGTTGAAAAATTGAG---ACCATGGTAGGGT
Andean lupine (Lupinus mutabilis) ITS2 Z72197.1 (26) CGTGCCAGG--------CACCAATCGGGGCGAATGTTGGCTTCCCGCGAGC-----AATGTCTCACGGTTGGTTGAAAACTGAG---TCCGCGGTGGAGG
blue lupine (L. angustifolius) ITS1 Z72202.1 (240) --
blue lupine (L. angustifolius) ITS2 Z72203.1 (26) CGTGCTAGG--------CACCAAGCGGGGCGAATGTTGGCTTCCCGCGAGC-----AATGTCTCACGGTTGGTTGAAAACTGAG---TCCGCGGTGGAGG
Andean lupine (Lupinus mutabilis) ITS1 Z72196.1 (240) --
yellow lupine (Lupinus luteus) ITS AF007478.1 (424) CGTGCCAGG--------CACGAAGCGGGGCGAATGTTGGCTTCCCGGGAGC-----AATGTCTCACGGTTGGTTGAAAACTGAG---TCCGCGGTGGAGG
yellow lupine (Lupinus luteus) ITS1 Z72206.1 (240) --
yellow lupine (Lupinus luteus) ITS DQ524249.1 (425) CGTGCCAGG--------CACGAAGCGGGGCGAATGTTGGCTTCCCGGGAGC-----AATGTCTCACGGTTGGTTGAAAACTGAG---TCCGCGGTGGAGG
yellow lupine (Lupinus luteus) ITS2 Z72207.1 (26) CGTGCCAGG--------CACGAAGCGGGGCGAATGTTGGCTTCCCGGGAGC-----AATGTCTCACGGTTGGTTGAAAACTGAG---TCCGCGGTGGAGG
blue lupine (L. angustifolius) ITS AF007477.1 (423) CGTGCTAGG--------CACCAAGCGGGGCGAATGTTGGCTTCCCGCGAGC-----AATGTCTCACGGTTGGTTGAAAACTGAG---TCCGCGGTGGAGG
blue lupine (L. angustifolius) ITS DQ524192.1 (423) CGTGCTAGG--------CACCAAGCGGGGCGAATGTTGGCTTCCCGCGAGC-----AATGTCTCACGGTTGGTTGAAAACTGAG---TCCGCGGTGGAGG
Andean lupine (Lupinus mutabilis) ITS AF007484.1 (425) CGTGCCAGG--------CACCAATCGGGGCGAATGTTGGCTTCCCGCGAGC-----AATGTCTCACGGTTGGTTGAAAACTGAG---TCCGCGGTGGAGG
Andean lupine (Lupinus mutabilis) ITS DQ524264.1 (425) CGTGCCAGG--------CACCAATCGGGGCGAATGTTGGCTTCCCGCGAGC-----AATGTCTCACGGTTGGTTGAAAACTGAG---TCCGCGGTGGAGG
white lupine (Lupinus albus) ITS AF007481.1 (424) CGTGCTAGG--------CACCAAGCGGGGCGAATGTTGGCTTCCCGTGAGC-----AATGTCTCACGGTTGGTTGAAAACTGAG---TCCGCGGTGGAGG
white lupine (Lupinus albus) ITS AY338946.1 (430) CGTGCTAGG--------CACCAAGCGGGGCGAATGTTGGCTTCCCGTGAGC-----AATGTCTCACGGTTGGTTGAAAACTGAG---TCCGCGGTGGAGG
white lupine (Lupinus albus) ITS1 Z72198.1 (240) --

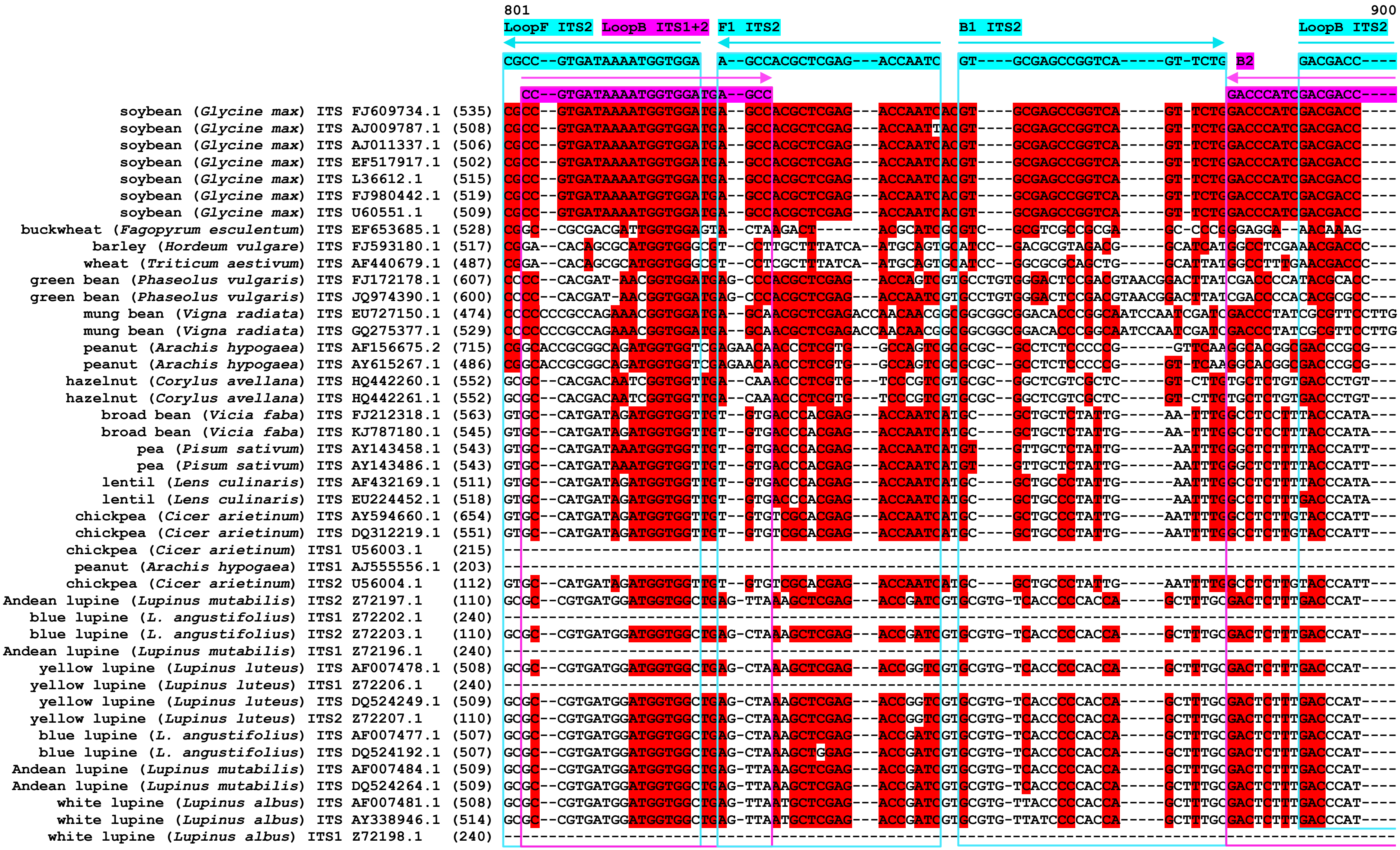

801
900
LoopF ITS2
LoopB ITS1+2
F1 ITS2
B1 ITS2
LoopB ITS2
B2
CGCC--GTGATAAAATGGTGGA
A--GCCACGCTCGAG---ACCAATC
GT----GCGAGCCGGTCA-----GT-TCTG
GACGACC----
CC--GTGATAAAATGGTGGATGA--GCC
GACCCATCGACGACC----
soybean (Glycine max) ITS FJ609734.1 (535) CGCC--GTGATAAAATGGTGGATGA--GCCACGCTCGAG---ACCAATCACGT----GCGAGCCGGTCA-----GT-TCTGGACCCATCGACGACC----
soybean (Glycine max) ITS AJ009787.1 (508) CGCC--GTGATAAAATGGTGGATGA--GCCACGCTCGAG---ACCAATTACGT----GCGAGCCGGTCA-----GT-TCTGGACCCATCGACGACC----
soybean (Glycine max) ITS AJ011337.1 (506) CGCC--GTGATAAAATGGTGGATGA--GCCACGCTCGAG---ACCAATCACGT----GCGAGCCGGTCA-----GT-TCTGGACCCATCGACGACC----
soybean (Glycine max) ITS EF517917.1 (502) CGCC--GTGATAAAATGGTGGATGA--GCCACGCTCGAG---ACCAATCACGT----GCGAGCCGGTCA-----GT-TCTGGACCCATCGACGACC----
soybean (Glycine max) ITS L36612.1 (515) CGCC--GTGATAAAATGGTGGATGA--GCCACGCTCGAG---ACCAATCACGT----GCGAGCCGGTCA-----GT-TCTGGACCCATCGACGACC----
soybean (Glycine max) ITS FJ980442.1 (519) CGCC--GTGATAAAATGGTGGATGA--GCCACGCTCGAG---ACCAATCACGT----GCGAGCCGGTCA-----GT-TCTGGACCCATCGACGACC----
soybean (Glycine max) ITS U60551.1 (509) CGCC--GTGATAAAATGGTGGATGA--GCCACGCTCGAG---ACCAATCACGT----GCGAGCCGGTCA-----GT-TCTGGACCCATCGACGACC----
buckwheat (Fagopyrum esculentum) ITS EF653685.1 (528) CGGC--CGCGACGATTGGTGGAGTA--CTAAGACT-------ACGCATCGCGTC---GCGTCGCCGCGA-----GC-CCCGGGAGGA--AACAAAG----
barley (Hordeum vulgare) ITS FJ593180.1 (517) CGGA--CACAGCGCATGGTGGGCGT--CCTTGCTTTATCA--ATGCAGTGCATCC--GACGCGTAGACG-----GCATCATGGCCTCGAAACGACCC---
wheat (Triticum aestivum) ITS AF440679.1 (487) CGGA--CACAGCGCATGGTGGGCGT--CCTCGCTTTATCA--ATGCAGTGCATCC--GGCGCGCAGCTG-----GCATTATGGCCTTTGAACGACCC---
green bean (Phaseolus vulgaris) ITS FJ172178.1 (607) CCCC--CACGAT-AACGGTGGATGAG-CCCACGCTCGAG---ACCAGTCGTGCCTGTGGGACTCCGACGTAACGGACTTATCGACCCCATACGCACC---
green bean (Phaseolus vulgaris) ITS JQ974390.1 (600) CCCC--CACGAT-AACGGTGGATGAG-CCCACGCTCGAG---ACCAATCGTGCCTGTGGGACTCCGACGTAACGGACTTATCGACCCCACACGCGCC---
mung bean (Vigna radiata) ITS EU727150.1 (474) CCCCCCCGCCAGAAACGGTGGATGA--GCAACGCTCGAGACCAACAACGGCGGCGGCGGACACCCGGCAATCCAATCGATCGACCCTATCGCGTTCCTTG
mung bean (Vigna radiata) ITS GQ275377.1 (529) CCCCCCCGCCAGAAACGGTGGATGA--GCAACGCTCGAGACCAACAACGGCGGCGGCGGACACCCGGCAATCCAATCGATCGACCCTATCGCGTTCCTTG
peanut (Arachis hypogaea) ITS AF156675.2 (715) CGGCACCGCGGCAGATGGTGGTCGAGAACAACCCTCGTG---GCCAGTCGCGCGC--GCCTCTCCCCCG------GTTCAAGGCACGGCGACCCGCG---
peanut (Arachis hypogaea) ITS AY615267.1 (486) CGGCACCGCGGCAGATGGTGGTCGAGAACAACCCTCGTG---GCCAGTCGCGCGC--GCCTCTCCCCCG-----GT-TCAAGGCACGGCGACCCGCG---
hazelnut (Corylus avellana) ITS HQ442260.1 (552) GCGC--CACGACAATCGGTGGTTGA--CAAACCCTCGTG---TCCCGTCGTGCGC--GGCTCGTCGCTC-----GT-CTTGTGCTCTGTGACCCTGT---
hazelnut (Corylus avellana) ITS HQ442261.1 (552) GCGC--CACGACAATCGGTGGTTGA--CAAACCCTCGTG---TCCCGTCGTGCGC--GGCTCGTCGCTC-----GT-CTTGTGCTCTGTGACCCTGT---
broad bean (Vicia faba) ITS FJ212318.1 (563) GTGC--CATGATAGATGGTGGTTGT--GTGACCCACGAG---ACCAATCATGC----GCTGCTCTATTG-----AA-TTTGGCCTCCTTTACCCATA---
broad bean (Vicia faba) ITS KJ787180.1 (545) GTGC--CATGATAGATGGTGGTTGT--GTGACCCACGAG---ACCAATCATGC----GCTGCTCTATTG-----AA-TTTGGCCTCCTTTACCCATA---
pea (Pisum sativum) ITS AY143458.1 (543) GTGC--CATGATAAATGGTGGTTGT--GTGACCCACGAG---ACCAATCATGT----GTTGCTCTATTG------AATTTGGGCTCTTTTACCCATT---
pea (Pisum sativum) ITS AY143486.1 (543) GTGC--CATGATAAATGGTGGTTGT--GTGACCCACGAG---ACCAATCATGT----GTTGCTCTATTG------AATTTGGGCTCTTTTACCCATT---
lentil (Lens culinaris) ITS AF432169.1 (511) GTGC--CATGATAGATGGTGGTTGT--GTGACCCACGAG---ACCAATCATGC----GCTGCCCTATTG------AATTTGGCCTCTTTTACCCATA---
lentil (Lens culinaris) ITS EU224452.1 (518) GTGC--CATGATAGATGGTGGTTGT--GTGACCCACGAG---ACCAATCATGC----GCTGCCCTATTG------AATTTGGCCTCTTTACCCATA---
chickpea (Cicer arietinum) ITS AY594660.1 (654) GTGC--CATGATAGATGGTGGTTGT--GTGTCGCACGAG---ACCAATCATGC----GCTGCCCTATTG-----AATTTTGGCCTCTTGTACCCATT---
chickpea (Cicer arietinum) ITS DQ312219.1 (551) GTGC--CATGATAGATGGTGGTTGT--GTGTCGCACGAG---ACCAATCATGC----GCTGCCCTATTG-----AATTTTGGCCTCTTGTACCCATT---
chickpea (Cicer arietinum) ITS1 U56003.1 (215) --
peanut (Arachis hypogaea) ITS1 AJ555556.1 (203) --
chickpea (Cicer arietinum) ITS2 U56004.1 (112) GTGC--CATGATAGATGGTGGTTGT--GTGTCGCACGAG---ACCAATCATGC----GCTGCCCTATTG-----AATTTTGGCCTCTTGTACCCATT---
Andean lupine (Lupinus mutabilis) ITS2 Z72197.1 (110) GCGC--CGTGATGGATGGTGGCTGAG-TTAAAGCTCGAG---ACCGATCGTGCGTG-TCACCCCCACCA-----GCTTTGCGACTCTTTGACCCAT----
blue lupine (L. angustifolius) ITS1 Z72202.1 (240) --
blue lupine (L. angustifolius) ITS2 Z72203.1 (110) GCGC--CGTGATGGATGGTGGCTGAG-CTAAAGCTCGAG---ACCGATCGTGCGTG-TCACCCCCACCA-----GCTTTGCGACTCTTTGACCCAT----
Andean lupine (Lupinus mutabilis) ITS1 Z72196.1 (240) --
yellow lupine (Lupinus luteus) ITS AF007478.1 (508) GCGC--CGTGATGGATGGTGGCTGAG-CTAAAGCTCGAG---ACCGGTCGTGCGTG-TCACCCCCACCA-----GCTTTGCGACTCTTTGACCCAT----
yellow lupine (Lupinus luteus) ITS1 Z72206.1 (240) --
yellow lupine (Lupinus luteus) ITS DQ524249.1 (509) GCGC--CGTGATGGATGGTGGCTGAG-CTAAAGCTCGAG---ACCGGTCGTGCGTG-TCACCCCCACCA-----GCTTTGCGACTCTTTGACCCAT----
yellow lupine (Lupinus luteus) ITS2 Z72207.1 (110) GCGC--CGTGATGGATGGTGGCTGAG-CTAAAGCTCGAG---ACCGGTCGTGCGTG-TCACCCCCACCA-----GCTTTGCGACTCTTTGACCCAT----
blue lupine (L. angustifolius) ITS AF007477.1 (507) GCGC--CGTGATGGATGGTGGCTGAG-CTAAAGCTCGAG---ACCGATCGTGCGTG-TCACCCCCACCA-----GCTTTGCGACTCTTTGACCCAT----
blue lupine (L. angustifolius) ITS DQ524192.1 (507) GCGC--CGTGATGGATGGTGGCTGAG-CTAAAGCTGGAG---ACCGATCGTGCGTG-TCACCCCCACCA-----GCTTTGCGACTCTTTGACCCAT----
Andean lupine (Lupinus mutabilis) ITS AF007484.1 (509) GCGC--CGTGATGGATGGTGGCTGAG-TTAAAGCTCGAG---ACCGATCGTGCGTG-TCACCCCCACCA-----GCTTTGCGACTCTTTGACCCAT----
Andean lupine (Lupinus mutabilis) ITS DQ524264.1 (509) GCGC--CGTGATGGATGGTGGCTGAG-TTAAAGCTCGAG---ACCGATCGTGCGTG-TCACCCCCACCA-----GCTTTGCGACTCTTTGACCCAT----
white lupine (Lupinus albus) ITS AF007481.1 (508) GCGC--CGTGATGGATGGTGGCTGAG-TTAATGCTCGAG---ACCGATCGTGCGTG-TTACCCCCACCA-----GCTTTGCGACTCTTTGACCCAT----
white lupine (Lupinus albus) ITS AY338946.1 (514) GCGC--CGTGATGGATGGTGGCTGAG-TTAATGCTCGAG---ACCGATCGTGCGTG-TTATCCCCACCA-----GCTTTGCGACTCTTTGACCCAT----
white lupine (Lupinus albus) ITS1 Z72198.1 (240) --

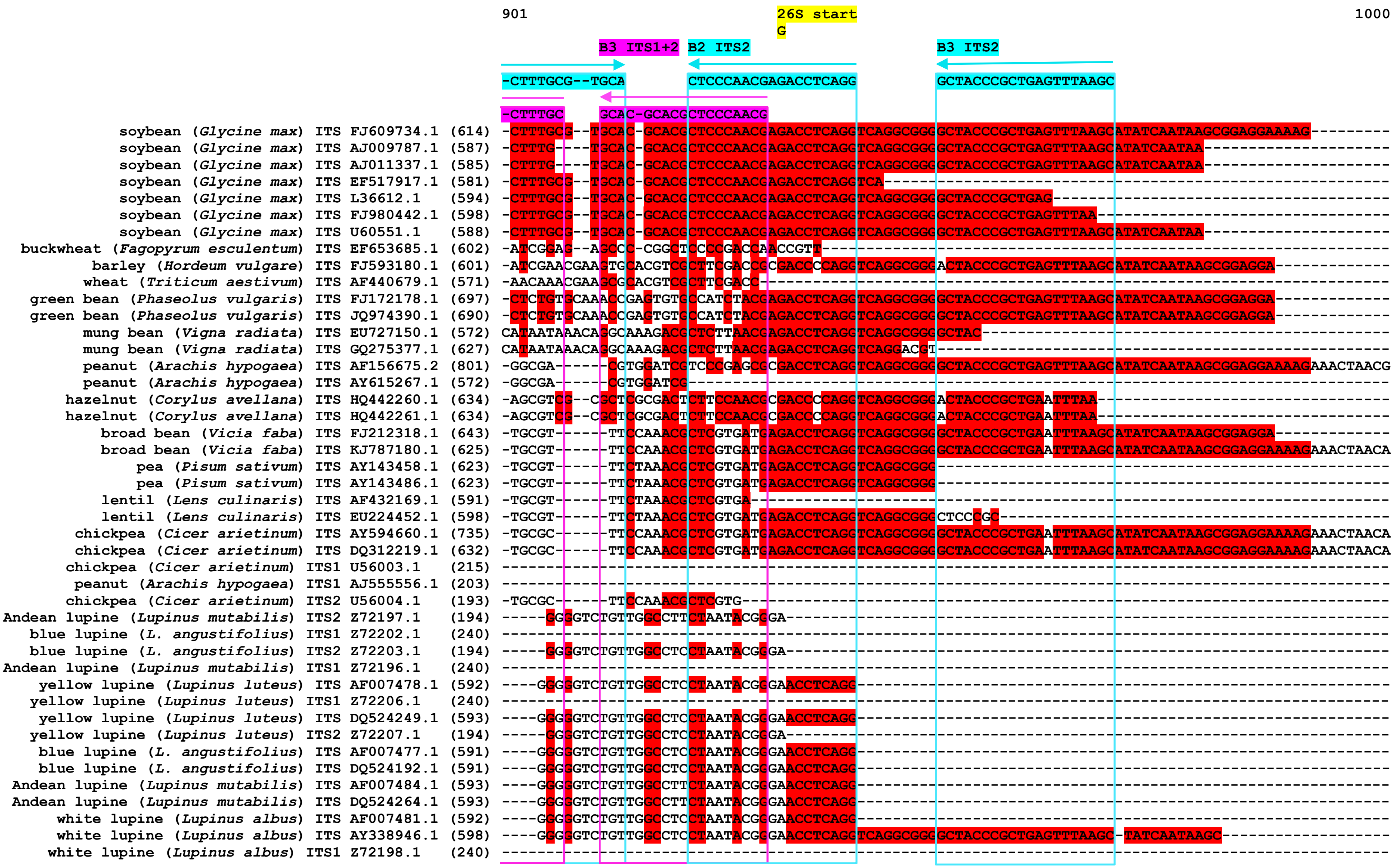

901
26S start
G
1000
B3 ITS1+2
B2 ITS2
B3 ITS2
-CTTTGCG--TGCA
CTCCCAACGAGACCTCAGG
GCTACCCGCTGAGTTTAAGC
-CTTTGC
GCAC-GCACGCTCCCAACG
soybean (Glycine max) ITS FJ609734.1 (614) -CTTTGCG--TGCAC-GCACGCTCCCAACGAGACCTCAGGTCAGGCGGGGCTACCCGCTGAGTTTAAGCATATCAATAAGCGGAGGAAAAG---------
soybean (Glycine max) ITS AJ009787.1 (587) -CTTTG----TGCAC-GCACGCTCCCAACGAGACCTCAGGTCAGGCGGGGCTACCCGCTGAGTTTAAGCATATCAATAA---------------------
soybean (Glycine max) ITS AJ011337.1 (585) -CTTTG----TGCAC-GCACGCTCCCAACGAGACCTCAGGTCAGGCGGGGCTACCCGCTGAGTTTAAGCATATCAATAA---------------------
soybean (Glycine max) ITS EF517917.1 (581) -CTTTGCG--TGCAC-GCACGCTCCCAACGAGACCTCAGGTCA---
soybean (Glycine max) ITS L36612.1 (594) -CTTTGCG--TGCAC-GCACGCTCCCAACGAGACCTCAGGTCAGGCGGGGCTACCCGCTGAG--------------------------------------
soybean (Glycine max) ITS FJ980442.1 (598) -CTTTGCG--TGCAC-GCACGCTCCCAACGAGACCTCAGGTCAGGCGGGGCTACCCGCTGAGTTTAA---------------------------------
soybean (Glycine max) ITS U60551.1 (588) -CTTTGCG--TGCAC-GCACGCTCCCAACGAGACCTCAGGTCAGGCGGGGCTACCCGCTGAGTTTAAGCATATCAATAA---------------------
buckwheat (Fagopyrum esculentum) ITS EF653685.1 (602) -ATCGGAG--AGCCC-CGGCTGCCCGACCAACCGTT--
barley (Hordeum vulgare) ITS FJ593180.1 (601) -ATCGAACGAAGTGCACGTCGCTTCGACCGCGACCCCAGGTCAGGCGGGACTACCCGCTGAGTTTAAGCATATCAATAAGCGGAGGA-------------
wheat (Triticum aestivum) ITS AF440679.1 (571) -AACAAACGAAGCGCACGTCGCTTCGACC---
green bean (Phaseolus vulgaris) ITS FJ172178.1 (697) -CTCTGTGCAAACCGAGTGTGCCATCTACGAGACCTCAGGTCAGGCGGGGCTACCCGCTGAGTTTAAGCATATCAATAAGCGGAGGA-------------
green bean (Phaseolus vulgaris) ITS JQ974390.1 (690) -CTCTGTGCAAACCGAGTGTGCCATCTACGAGACCTCAGGTCAGGCGGGGCTACCCGCTGAGTTTAAGCATATCAATAAGCGGAGGA-------------
mung bean (Vigna radiata) ITS EU727150.1 (572) CATAATAAACAGGCAAAGACGCTCTTAACGAGACCTCAGGTCAGGCGGGGCTAC--
mung bean (Vigna radiata) ITS GQ275377.1 (627) CATAATAAACAGGCAAAGACGCTCTTAACGAGACCTCAGGTCAGGACGT---
peanut (Arachis hypogaea) ITS AF156675.2 (801) -GGCGA------CGTGGATCGTCCCGAGCGCGACCTCAGGTCAGGCGGGGCTACCCGCTGAGTTTAAGCATATCAATAAGCGGAGGAAAAGAAACTAACG
peanut (Arachis hypogaea) ITS AY615267.1 (572) -GGCGA------CGTGGATCG---
hazelnut (Corylus avellana) ITS HQ442260.1 (634) -AGCGTCG--CGCTCGCGACTCTTCCAACGCGACCCCAGGTCAGGCGGGACTACCCGCTGAATTTAA---------------------------------
hazelnut (Corylus avellana) ITS HQ442261.1 (634) -AGCGTCG--CGCTCGCGACTCTTCCAACGCGACCCCAGGTCAGGCGGGACTACCCGCTGAATTTAA---------------------------------
broad bean (Vicia faba) ITS FJ212318.1 (643) -TGCGT------TTCCAAACGCTCGTGATGAGACCTCAGGTCAGGCGGGGCTACCCGCTGAATTTAAGCATATCAATAAGCGGAGGA-------------
broad bean (Vicia faba) ITS KJ787180.1 (625) -TGCGT------TTCCAAACGCTCGTGATGAGACCTCAGGTCAGGCGGGGCTACCCGCTGAATTTAAGCATATCAATAAGCGGAGGAAAAGAAACTAACA
pea (Pisum sativum) ITS AY143458.1 (623) -TGCGT------TTCTAAACGCTCGTGATGAGACCTCAGGTCAGGCGGG---
pea (Pisum sativum) ITS AY143486.1 (623) -TGCGT------TTCTAAACGCTCGTGATGAGACCTCAGGTCAGGCGGG---
lentil (Lens culinaris) ITS AF432169.1 (591) -TGCGT------TTCTAAACGCTCGTGA--
lentil (Lens culinaris) ITS EU224452.1 (598) -TGCGT------TTCTAAACGCTCGTGATGAGACCTCAGGTCAGGCGGGCTCCCGC--
chickpea (Cicer arietinum) ITS AY594660.1 (735) -TGCGC------TTCCAAACGCTCGTGATGAGACCTCAGGTCAGGCGGGGCTACCCGCTGAATTTAAGCATATCAATAAGCGGAGGAAAAGAAACTAACA
chickpea (Cicer arietinum) ITS DQ312219.1 (632) -TGCGC------TTCCAAACGCTCGTGATGAGACCTCAGGTCAGGCGGGGCTACCCGCTGAATTTAAGCATATCAATAAGCGGAGGAAAAGAAACTAACA
chickpea (Cicer arietinum) ITS1 U56003.1 (215) --
peanut (Arachis hypogaea) ITS1 AJ555556.1 (203) --
chickpea (Cicer arietinum) ITS2 U56004.1 (193) -TGCGC------TTCCAAACGCTCGTG---
Andean lupine (Lupinus mutabilis) ITS2 Z72197.1 (194) -----GGGGTCTGTTGGCCTTCTAATACGGGA--
blue lupine (L. angustifolius) ITS1 Z72202.1 (240) --
blue lupine (L. angustifolius) ITS2 Z72203.1 (194) -----GGGGTCTGTTGGCCTCCTAATACGGGA--
Andean lupine (Lupinus mutabilis) ITS1 Z72196.1 (240) --
yellow lupine (Lupinus luteus) ITS AF007478.1 (592) ----GGGGGTCTGTTGGCCTCCTAATACGGGAACCTCAGG--
yellow lupine (Lupinus luteus) ITS1 Z72206.1 (240) --
yellow lupine (Lupinus luteus) ITS DQ524249.1 (593) ----GGGGGTCTGTTGGCCTCCTAATACGGGAACCTCAGG--
yellow lupine (Lupinus luteus) ITS2 Z72207.1 (194) -----GGGGTCTGTTGGCCTCCTAATACGGGA--
blue lupine (L. angustifolius) ITS AF007477.1 (591) ----GGGGGTCTGTTGGCCTCCTAATACGGGAACCTCAGG--
blue lupine (L. angustifolius) ITS DQ524192.1 (591) ----GGGGGTCTGTTGGCCTCCTAATACGGGAACCTCAGG--
Andean lupine (Lupinus mutabilis) ITS AF007484.1 (593) ----GGGGGTCTGTTGGCCTTCTAATACGGGAACCTCAGG--
Andean lupine (Lupinus mutabilis) ITS DQ524264.1 (593) ----GGGGGTCTGTTGGCCTTCTAATACGGGAACCTCAGG--
white lupine (Lupinus albus) ITS AF007481.1 (592) ----GGGGGTCTGTTGGCCTCCTAATACGGGAACCTCAGG--
white lupine (Lupinus albus) ITS AY338946.1 (598) ----GGGGGTCTGTTGGCCTCCTAATACGGGAACCTCAGGTCAGGCGGGGCTACCCGCTGAGTTTAAGC-TATCAATAAGC-------------------
white lupine (Lupinus albus) ITS1 Z72198.1 (240) --

Figure S3. Sequence alignment of the mitochondrial genome of soybean (*Glycine max*; nucleotides 205217-205754), sequence of the open reading frame 160b (ORF160b; nucleotides 205230-205712). Red: homologous nucleotides in comparison with the reference sequence of soybean (*Glycine max*, GenBank acc. No. JX463295.1); yellow: start and end of the ORF160b of soybean (*Glycine max*, acc. No. JX463295.1); green: newly designed LAMP primers targeting the ORF160b of soybean (*Glycine max*, GenBank acc. No. JX463295.1)

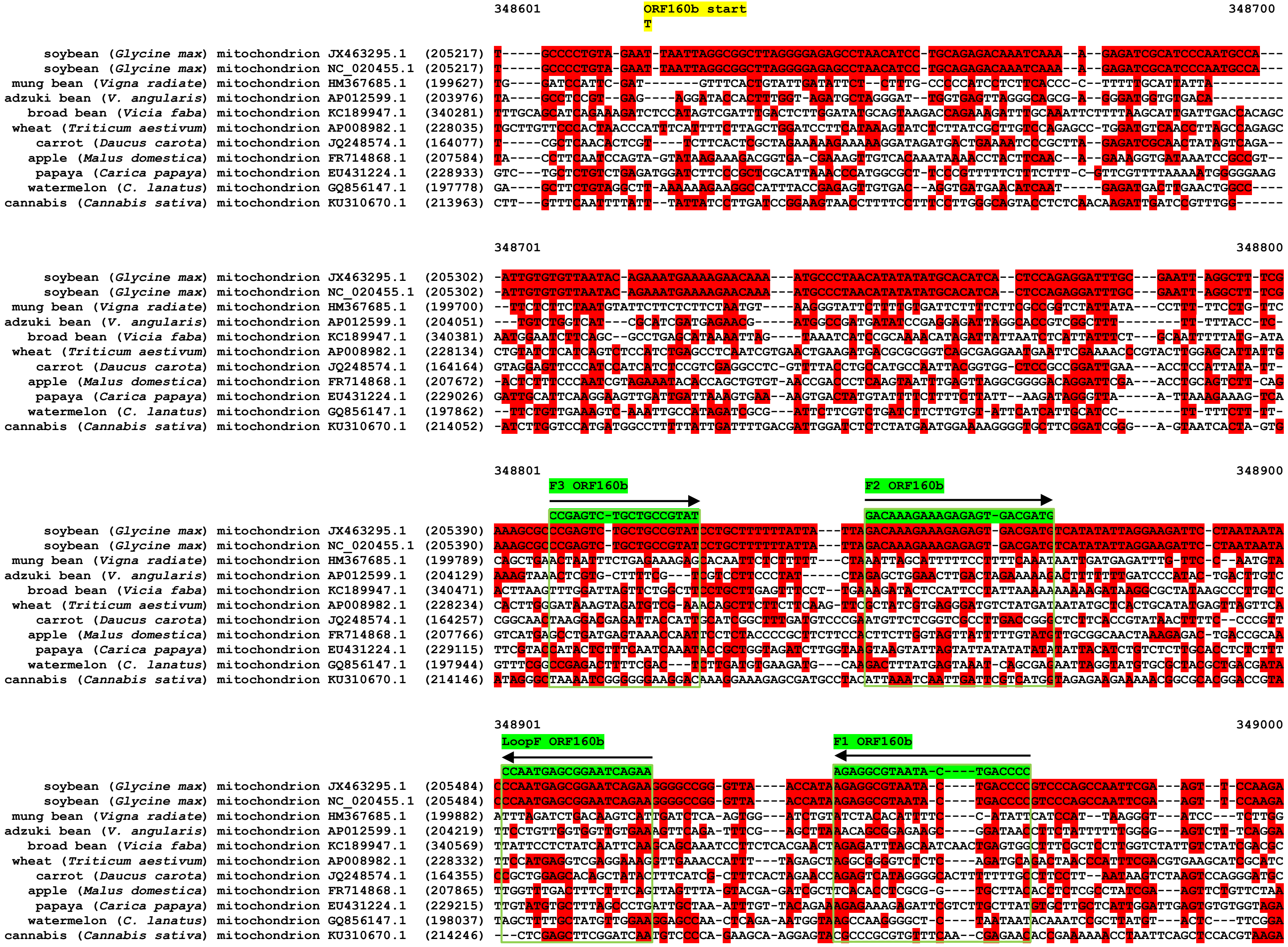

```
                                                                       349001                                                                                              349100
                                                                                         B1 ORF160b                           LoopB ORF160b
                                                                                         TCTGATAGATAGTGGCAAACATTAG            CAAGATATAGAAGACTATT-AGCCCG              AT
   soybean (Glycine max) mitochondrion JX463295.1    (205568)  TAAATTTG------------CTATTCTGATAGATAGTGGCAAACATTAGATTGTT-GTATCCCAAGATATAGAAGACTATT-AGCCCGAAAATTAGATTAT
   soybean (Glycine max) mitochondrion NC_020455.1   (205568)  TAAATTTG------------CTATTCTGATAGATAGTGGCAAACATTAGATTGTT-GTATCCCAAGATATAGAAGACTATT-AGCCCGAAAATTAGATTAT
  mung bean (Vigna radiate) mitochondrion HM367685.1 (199965)  TGTTACCT------------ATAGTTCTAA-G--AGTGGTTGTACTCTCTATCTT-TTTAACCACAAGTTCTTTCCAAT------CGGTTCGAAAAGGATA
adzuki bean (V. angularis) mitochondrion AP012599.1  (204307)  T-------------------CGATTCAAAAGGCCAGTGAAAGTACGCGGACGGAT-AAATCCAACAAAACCACACCGTA-----CCGGGCCAGTAGACCTT
  broad bean (Vicia faba) mitochondrion KC189947.1   (340669)  CCTTTTTT------------CTTTATTTTTGTTCATCCTTTGACTCAGCAAGGGAACGGAAAGCAATTCAATGGCAACTTGAAGCAGAATGTCCGTCTTT
 wheat (Triticum aestivum) mitochondrion AP008982.1  (228425)  GGCTTGTGATCCTA--AATCTAGTGATTGAT-TATTGACTTGTCAAGGGCAGGTTGGTGTCTTAATGTGGAGGAATTCCGTACCTAAGATAAGAAGTTTC
   carrot (Daucus carota) mitochondrion JQ248574.1   (164452)  CCCCTATG-----------TTTTTTCGTGGAGCGCATAATTTTCCTTAATCGGCGAGAGTATATATACATTTCACTGGCAAG--AGCATGATTGAGGGCTTTC
   apple (Malus domestica) mitochondrion FR714868.1  (207955)  AACCTCTT------------CCGAGAACTTGTATAGAGAAGGATTTCCCGCAGAG---CTGCAGTTCTTCCATACCAACTT-AGCTGCCCGGCGCTGCTAT
    papaya (Carica papaya) mitochondrion EU431224.1  (229313)  GACTTTTGACCGAACTGCTCTTTGCTTCACCAAATATGCTGTTCTCTTCTATTTATTATAGAATCATTTTTAAGTCACTT-CGTTAGTGTTCCGGGAAAG
    watermelon (C. lanatus) mitochondrion GQ856147.1  (198124)  GGCTTGTG------------CGTCCCGACAGGAAAAAGAATTTCTTACTACTGTTTGCCTTGTGCATGACAATACACAA----GCAAGACTGTTCGTCTAG
 cannabis (Cannabis sativa) mitochondrion KU310670.1 (214339)  TAGCCAAA---------GTACGGTTGAGCAATCGACATGATATATTTGCTCACAT-TCCGGGCGAAGGTCATAATTCGCAG-GAACATTCTATGGTGTTAA
```

```
                                                                       349101                                                ORF160b end                       349200
                                                                       B2 ORF160b                         B3 ORF160b         T
                                                                       GAATAGATGGAATAGCAGCAA              TGCGTGGA-ACTCAATCTCAT
   soybean (Glycine max) mitochondrion JX463295.1    (205655)  GAATAGATGGAATAGCAGCAAAAAGCATCTTTCTAGCTTGCGTGGA-ACTCAATCTCATTTAGTTAGAAAGGTTCTATCTA--TCTTTCAGCAACTG-AA
   soybean (Glycine max) mitochondrion NC_020455.1   (205655)  GAATAGATGGAATAGCAGCAAAAAGCATCTTTCTAGCTTGCGTGGA-ACTCAATCTCATTTAGTTAGAAAGGTTCTATCTA--TCTTTCAGCAACTG-AA
  mung bean (Vigna radiate) mitochondrion HM367685.1 (200044)  AGTTACATTCTCTATCACACTTCTGGATGGGTCGGCGTACTGC----TCTTTATACCATTCTGGAGTTAT----AACACT---TCTACCCGCTCCCT-TG
adzuki bean (V. angularis) mitochondrion AP012599.1  (204383)  CTTTCTCTTCTCTTAAAGGCTGTCTCCTGCCCCCAACTAGCGAGCG-GCTATAACCGAT---GGGCGCATGGCGACCACTAC-TCTACCCGAAAACT-AG
  broad bean (Vicia faba) mitochondrion KC189947.1   (340758)  CTTTATGCTATGTAAATTAA-AGAATCTCTGGCCTCCTGGCAAAGACCCTTCCTCGAAAAATCTTTTATCT--ATTCTCT---TCGACCCCCACTTTTTA
 wheat (Triticum aestivum) mitochondrion AP008982.1  (228522)  ATTTCATGCCTACCGTAGTCCCTATTTTATTGGGCATAGTCCCCTTACCTACTCCCGAGA--GTAGAAAATCAACATTTATGTCGTTCTTAATAAGAAA
   carrot (Daucus carota) mitochondrion JQ248574.1   (164540)  TACTGTGGTCTCTAACTCTTTAATTGGTCATTGGCTCTACCTAG---ACTAGATGAGATGCCGGTGCCATTAAACTAGCTGCCAGAACGAGTTCAAGAGA
   apple (Malus domestica) mitochondrion FR714868.1  (208040)  TGGCATAACAACCGGTACACCATAGGTTGGCCCAACCCAGTCC-------TC-TCGTACTAGGGTTGGCTC---CTCGCAGT-TCTCCCTTTAACACCAA
    papaya (Carica papaya) mitochondrion EU431224.1  (229412)  CAAAAGATTGAACAAAAG---ATTGACTATATTAAACAAGCAAGCA-CGAAAATCAAAGCT-GTTCTTTTTA-TATAAGTAA-CGGCCCAGTACATTGAA
    watermelon (C. lanatus) mitochondrion GQ856147.1  (198209)  TGGTATCCTGTATCTTA---TATAAGATCTGTGTCCTTACCCT----A-TGCATGGTATTTCGTTAGTGA---AAGATCTAT-TCTAGCCGTTCTTGCTA
 cannabis (Cannabis sativa) mitochondrion KU310670.1 (214429)  TAAGAGGAGGTAGAGTGAAAGATTTGCCAGGTGTGAAATCCCATTGTATTCGAGGAGTCAAAGATTTGCTGGGAATTCCGGATCGAAGAAGAGGCAG--A
```
